# Vitamin D protects spermatogonia and Sertoli cells from heat stress damage by inhibiting NLRP3

**DOI:** 10.3389/fped.2024.1495310

**Published:** 2025-01-07

**Authors:** Han Chu, Qi-Fei Deng, Yuan Fang

**Affiliations:** ^1^ The Department of Pediatric Urology Surgery, Anhui Provincial Children’s Hospital, Hefei, Anhui, China; ^2^ Pathology Department, Anhui Provincial Children’s Hospital, Hefei, Anhui, China

**Keywords:** cryptorchidism, spermatogonia, sertoli cell, vitamin D, NLRP3

## Abstract

**Introduction:**

Cryptorchidism can damage cells in the cryptorchid testes due to elevated local temperatures, potentially impacting the fertility of the child in adulthood. Research indicates that vitamin D enhances sperm quality in adult males. This study aimed to explore whether vitamin D inhibits NLRP3 activation, thus helping to mitigate heat stress damage to testicular spermatogenic and Sertoli cells.

**Materials and methods:**

Five cases of normal testicular tissue adjacent to a tumor (testis removed due to tumorous growth) and five cases of atrophied cryptorchid testicular tissue (testis removed) were analyzed for immunohistochemistry to determine NLRP3 expression in cryptorchid tissue. In Phase I, spermatogonia (GC-1) and Sertoli cells (TM4) were separated into blank and heat stress groups. Apoptosis, inflammatory factor levels, and the expression of Bcl-2 and NLRP3 genes and proteins were measured at 2, 6, and 10 h after heat stress treatment. In Phase II, the cells were re-cultured and divided into three groups: heat stress, siRNA + heat stress, and VD + heat stress. After 10 h, the apoptosis, inflammatory factor levels, and gene and protein expressions of Bcl-2 and NLRP3 were reassessed in each group.

**Results:**

Immunohistochemistry indicated NLRP3 expression in cryptorchid tissue. Phase I, extending heat stress duration led to increased apoptosis in spermatogonia (GC-1) and testicular Sertoli cells (TM4), heightened levels of inflammatory factors, reduced BCL-2 expression, and elevated NLRP3 expression compared to the control group. Phase II, both the siRNA + heat stress and VD + heat stress groups showed decreased apoptosis in spermatogonia and Sertoli cells, lower inflammatory factor levels, increased BCL-2 expression, and decreased NLRP3 expression compared to the heat stress-only group, with statistically significant differences (*P* < 0.05).

**Conclusions:**

This is the first time we found the expression of NLRP3 in cryptorchidism. Vitamin D can inhibit the expression of NLRP3 and reduce the damage of heat stress on testicular spermatogenic cells and Sertoli cells, and play a protective role for testicular spermatogenic cells and Sertoli cells. This provides a theoretical basis for preserving testicular function during the “treatment gap” in boys with cryptorchidism who have not received surgical treatment.

## 1 Introduction

Cryptorchidism is one of the congenital anomalies of the genitourinary tract in children, with a prevalence of approximately 1%–9% in newborns (1). The optimal time for surgery is 6–12 months (after correction for gestational age), and if the optimal time for treatment is missed, fertility decreases in adulthood along with an increased chance of developing testicular tumors (2).

In the boy's early childhood, Sertoli cells as a part of the spermatogenic tubules, are harbours for spermatogonia and are phagocytic, non-professional antigen presenting cells (3). Damage to both types of cells can affect sperm production in adulthood.

Under normal conditions, the temperature of the scrotum is usually slightly lower than the body temperature by about 2–7 degrees Celsius. This lower temperature helps maintain normal testicular function and sperm production. Impairment of testicular spermatogonia and Sertoli cells due to high scrotal temperatures has been demonstrated (4). However, children with cryptorchidism within 6–12months of age, especially those with bilateral or high-positioned cryptorchidism, have a small chance of self-descent to the scrotum, and there is persistent heat stress damage to testicular spermatogonia and Sertoli cells (5). Studies have shown that even in children who undergo surgery within this timeframe, the damage to the testicular spermatogonia and Sertoli cells is not restored (6). It is still possible to recover from transient damage to spermatogonia and Sertoli cells from heat stress, but not from prolonged heat stress (7). So how to reduce the degree of damage to testicular spermatogonia in this stage of life is worth studying.

Vitamin D (VD) supplementation in children may improve and promote foetal growth and development, and effectively prevent and treat the onset and progression of inflammatory diseases (8, 9). Hirai et al. conducted an animal experiment, feeding cryptorchid mice with vitamin D for 4 weeks, and found that vitamin D can promote spermatogenesis by upregulating certain specific genes in Sertoli cells (10). Vitamin D supplementation has been shown to help improve fertility and sperm viability in adult men (11).

Bazrafkan et al. found that in COVID-19 patients, the inflammatory response caused by SARS-CoV-2 leads to high expression of NLRP3 in testicular tissue, resulting in impaired sperm production and an increased risk of infertility. It is believed that inflammasome inhibitors may be appropriate candidates for preventing and improving fertility (12). Walenta et al. used AROM + mice (an animal model of male infertility) to explore NLRP3's role in male infertility in testes. They found NLRP3 transcriptional levels were up at 2, 5, and 10 months of age in mice testes, with a significant increase at 5 and 10 months. They confirmed that NLRP3's sterile inflammation might be involved in the pathogenesis of male infertility (13). NLRP3 has been shown to be expressed in testicular spermatogonial cells (GC-1) and Sertoli cells (TM4), and its overexpression under infection or immune stress can lead to dyspermatogenesis and male infertility (14, 15). It was suggested that NLRP3 might be a new idea and target for treating varicocele and testicular transplantation (16, 17). Wang et al. (18) divided mice into control and heated groups to study the effect of extreme heat on acute kidney injury. They found that activation of renal NLRP3 inflammasome and increased cytokines in the heated group led to renal impairment, suggesting a link between extreme heat and acute kidney injury via NLRP3 inflammasome activation. This confirms that the inflammatory response induced by high expression of NLRP3 can be triggered by heat stress.

However, there is no research on how to preserve the function of the undescended testis in children with cryptorchidism who have not undergone surgery. In this study, we conducted *in vitro* cell experiments to investigate whether heat stress leads to the overexpression of NLRP3 in spermatogonia and Sertoli cells of undescended testicular, and to see if vitamin D can inhibit the expression of NLRP3 triggered by heat stress, thus potentially alleviating the damage caused by heat stress to spermatogonia (GC-1) and Sertoli cells (TM4). To provide a theoretical basis and new insights for preserving testicular function in children with cryptorchidism without surgical intervention.

## 2 Materials and methods

Our experiment consisted of two parts: the organize experiments and cellular experiments, the latter of which has two parts.

### 2.1 Organize experiments: immunohistochemistry and confocal microscopy

With the appropriate approval from the Institute Review Board (IRB) and the Ethics Committee of Anhui Provincial Children's Hospital (approval no. eyrc012), we conducted a study from December 2021 to December 2023 involving children who underwent orchiectomy for testicular tumors and testicular remnant resection for nonpalpable cryptorchidism at our hospital. For the normal control group, we selected five cases of para-tumoral normal testicular tissues (without infiltration of tumor cells), and for the cryptorchidism group, we selected the residual testicular tissues from orchiectomy due to testicular regression syndrome, and chose five cases of pathological tissues containing seminiferous tubules as cryptorchid tissues. Immunohistochemistry was employed to confirm the expression of NLRP3 in both tissue samples.

Frozen blocks of five cryptorchid tissue specimens and five normal testicular tissue specimens (from tumor-free areas) were sectioned transversely at 10 μm, mounted on SuperFrost Plus slides, and fixed in 10% buffered formalin for 5 min. The sections underwent cell membrane permeabilization with 1% Triton X-100 for 20 min at room temperature. After blocking with 10% bovine serum albumin for 30 min to prevent nonspecific binding, the sections were incubated overnight at 4°C with a mixture of primary antibodies against NLRP3 (Cell Signaling Technology, USA, Rabbit, #D4D8T, 1:50) diluted in phosphate-buffered saline containing 1% bovine serum albumin. Following this, sections were washed in phosphate-buffered saline with 0.05% Tween and incubated with secondary antibodies (Roche, Swiss, ultraview HRP multimer, 253-4290) for 1 h at room temperature. They were then stained with hematoxylin for 1–2 min, rinsed with tap water or PBS, differentiated with 1% hydrochloric acid alcohol for 3 s, and rinsed again with tap water for 3 min. Dehydration proceeded through 70% alcohol for 1 min, 80% alcohol for 1 min, 95% alcohol for 2 min, absolute alcohol for 4 min, and xylene I and II for 3 min each. After air-drying the slides, a suitable amount of neutral gum was added, covered with a coverslip, and any air bubbles were removed. The slides were allowed to dry for half a day before being photographed under a microscope.

### 2.2 Cellular experiments

#### 2.2.1 Cell line and reagents

The GC-l cell lines and TM4 cell lines were kindly provided by Jiangsu Aidisheng Biological Technology Co. LTD. and the passages of these cell lines used for the experiments were approximately 17-25. 1,25-(OH)2D3 provided by Jiangsu Meibiao Biotechnology Co. LTD. DMEM/F12 medium and fetal bovine serum were purchased by Gibco (USA). Primary antibody NLRP3, β-actin and Bcl-2 were purchased from Cell Signaling Technology. The Cell Counting Kit 8 (CCK-8) solution is from the Nanjing Jiancheng Bioengineering Institute (nanjing jiancheng, China). Flow cytometry assay kit purchased from BD company. ELISA kit purchased from Wuhan Huamei. NLRP3-siRNA was constructed by Gima Pharmaceutical Technology Co. LTD (Shanghai, China). The Trizol reagent and Prime Script reverse transcriptase reagent kit are from TaKaRa Biotechnology.

### 2.3 Experimental methods

1) **Flow Cytometry:** Cellular apoptosis was assessed using flow cytometry (BD, FACSCalibur, USA) with fluorochrome-labeled caspase inhibitors (FLICA) and propidium iodide (PI) added to GC-1 cells. Apoptosis was identified by the presence of PI (+) and FLICA (+) after staining, aligning with the TM4 apoptosis assays.
2) **ELISA:** TNF-α and IL-1β levels in the culture medium were quantified using enzyme-linked immunosorbent assay (ELISA) kits (MM-0132M1, MM-0040M1, Jiangsu Meibiao Biotechnology Co. Ltd, China), following the manufacturer's instructions.
3) **Quantitative reverse transcription-polymerase chain reaction (qRT-PCR)** Total RNA was extracted using TRIzol reagent (Invitrogen) following the manufacturer's protocol. It was then reverse transcribed with the Easy RT-PCR kit (LS1040, Promega, Madison, WI, USA) as instructed. β-actin served as an internal control. Real-time PCR assays were conducted with Power SYBR Green Master Mix and an ABI 7,300 real-time PCR detection system (both from Applied Biosystems, USA). The RT-PCR primer sequences were: NLRP3 forward 5′-AACATGCCCAAGGAGGAAGA-3′, reverse 5′-GGCTGTTCACCAATCCATGA-3′. Actin forward 5′-CTACAATGAGCTGCGTGTGGC-3′, reverse 5′-CAGGTCCAGACGCAGGATGGC-3′. All primers were synthesized by TIANYI HUIYUAN (Beijing, China). Gene expression fold changes were calculated using the comparative threshold cycle (Ct) method with the formula 2^-(ΔΔCt).
4) **Western Blotting:** Proteins from GC-1 and TM4 cells were separated using SDS-PAGE and transferred to nitrocellulose membranes. The Pierce BCA Protein Assay Kit (23,225, Thermo Scientific, USA) was employed for quantitative protein analysis. The membranes were blocked with 5% skim milk for 2 h and then incubated with primary antibodies against NLRP3 (ab263899, rabbit, Abcam), Bcl-2 (26593-1-AP, rabbit, Proteintech), and β-Actin (bs-0016R, rabbit, BIOSS). After rinsing three times with TBST, the membranes were incubated with sheep secondary antibodies at room temperature for 2 h. β-Actin served as the endogenous control. Semi-quantitative analysis of band gray values was performed using ImageJ software 1.49v (National Institutes of Health, Bethesda, MD, USA). The target protein to actin gray value ratio was calculated for quantification, and statistical analysis was conducted on multiple measurements.

### 2.4 Cell culture and grouping

GC-1 and TM4 cells were regularly cultured in DMEM/F12 (Gibco, USA) with 10% (v/v) FBS, 100 u/ml penicillin, and 100 mg/ml streptomycin in a humidified 37°C incubator with 5% CO2% and 95% air.

#### 2.4.1 Phase I

**Figure 1:**
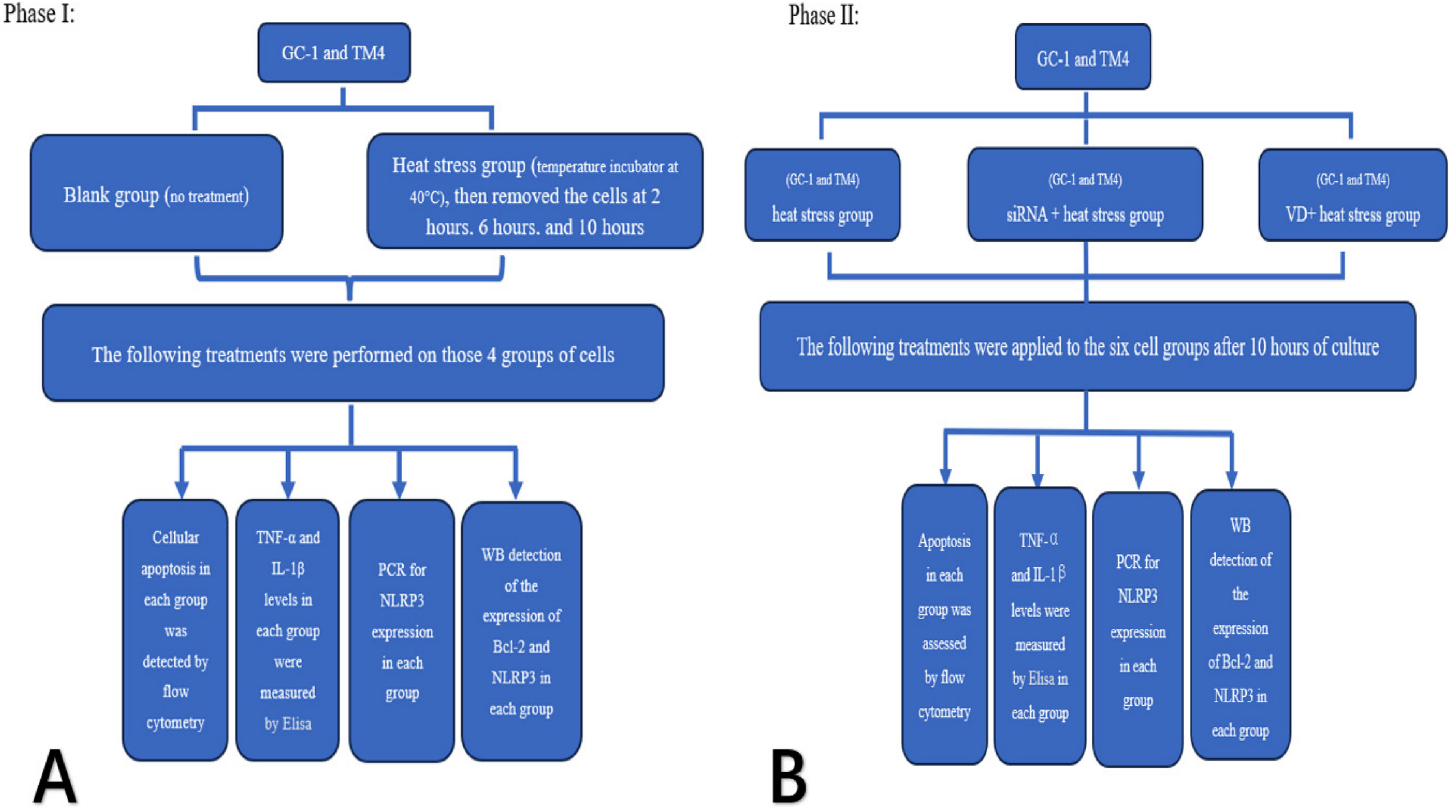
Experimental flow chart.

The two cell lines were divided into a control group and a heat stress group. The heat stress group was cultured at 40°C, and cells were extracted after being recovered at 37°C for 6 h at different time points (2, 6, and 10 h). Using the aforementioned experimental methods, we tested the four groups for cell apoptosis, inflammatory factor concentration (TNF-α and IL-1β), and the expression levels of Bcl-2 and NLRP3 (see Figure 1A).

#### 2.4.2 Phase II

One group of each of the two cell types was taken, and siRNA was used to silence the NLRP3 gene of both cell types as the siRNA + heat stress group.

#### 2.4.3 siRNA transfection

GC-1 and TM4 cells were separately p laced in RPMI 1,640 medium containing 10% fetal bovine serum, 100 U/ml penicillin, and 100 µg/ml streptomycin, and cultured under 5% CO2, 37°C humidity conditions. Cells in the logarithmic growth phase were seeded in P30 cell culture dishes after adjusting the cell concentration with trypsin digestion. When the cells reached 70%–80% confluence, siRNA transfection was performed according to the steps of the Lipofectamine 2,000 (Invitroge, USA.) transfection reagent kit. The cells were then continued to be cultured. The medium was changed 4–6 h after transfection, and the next step of the experiment was carried out according to the experimental requirements.

One group of each of the two cell types was taken and cultured by adding VD [1, 25-(OH)2D3, 30 ng/ml] as the VD + heat stress group.

The blank group was treated with heat stress only and served as the heat stress group.

Heat stress group, siRNA + heat stress group and VD + heat stress groups of two cell types were cultured at 40°C for 10 h (total six groups), and then recovered at 37°C for 6 h. Cells of each groups were extracted and tested for apoptosis, inflammatory factor concentration (TNF-α and IL-1β), and expression of Bcl-2 and NLRP3 by using the above method (see Figure 1B).

### 2.5 Statistical analysis

Data are presented as mean ± standard deviation (S.D.). Group differences were analyzed using *t*-tests or ANOVA with the SPSS 16.0 software, considering *P* < 0.05 as statistically significant.

## 3 Results

### 3.1 NLRP3 is expressed in cryptorchid tissues

**Figure 2:**
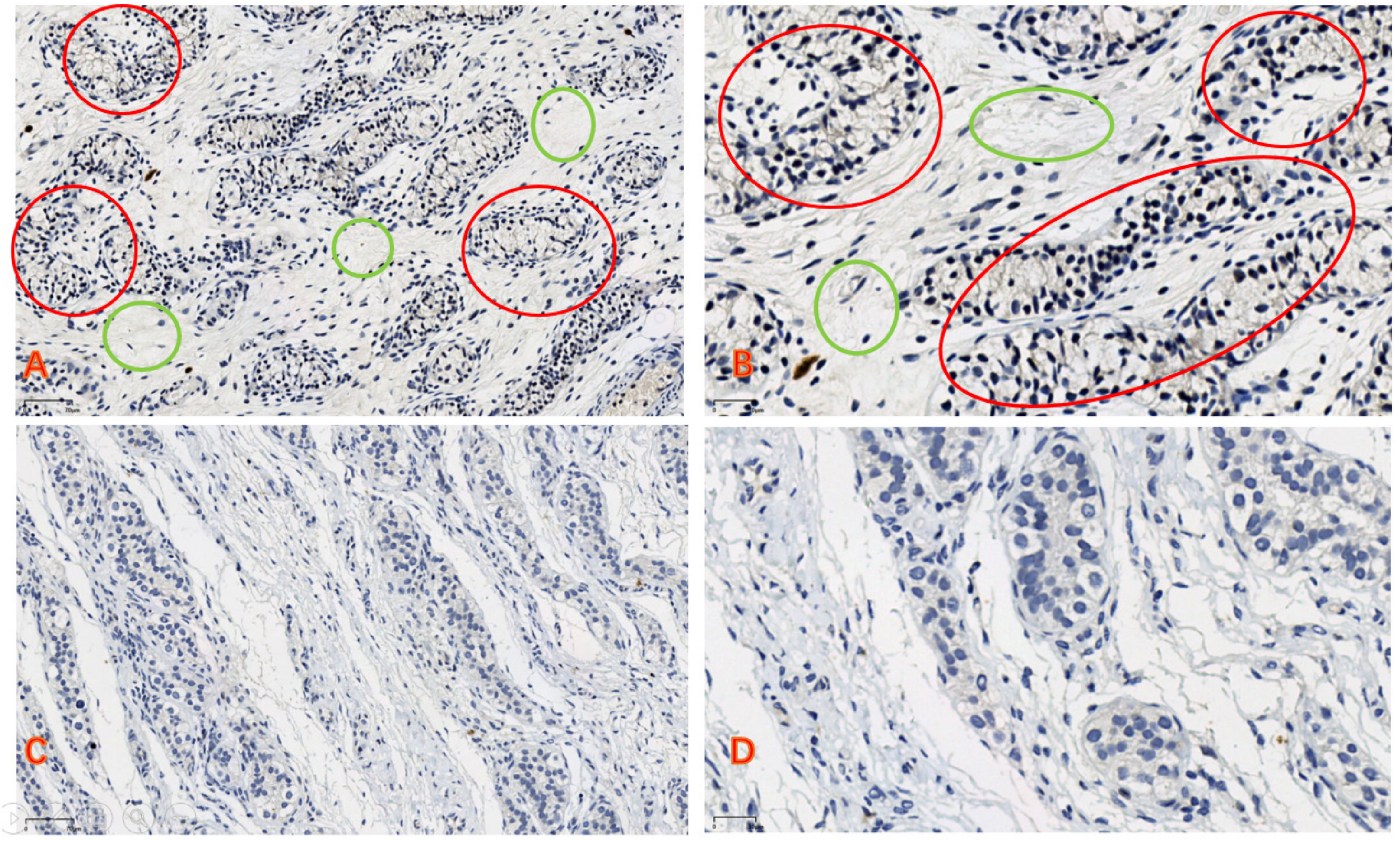
**(A,B)** In the cryptorchid tissue, the reproductive cells are small in size, and there is a significant increase in connective tissue (green circle). The expression of NLRP3 can be observed (red circle). **(C,D)** In normal testicular tissue, the reproductive cells develop well, there is relatively less connective tissue, and no expression of NLRP3 is observed **(A,C)** bars = 70 μm, **(B,D)** bars = 30 μm).

We used a fully automated digital slide scanner (UNIC, China) to scan the samples into digital slides, which were then opened and captured using the iViewer software. Figures A and C are magnified 100 times, while Figures B and D are magnified 200 times. The tubular structures represent seminiferous tubules, where the majority consist of short spindle-shaped support cells, with a minority being larger germ cells with clear cytoplasm. We assessed the expression levels based on the staining intensity of the slides. NLRP3 is weakly expressed in the perinuclear cytoplasm (Figure 2).

### 3.2 Heat stress increases apoptosis in the two types of cells

**Figure 3:**
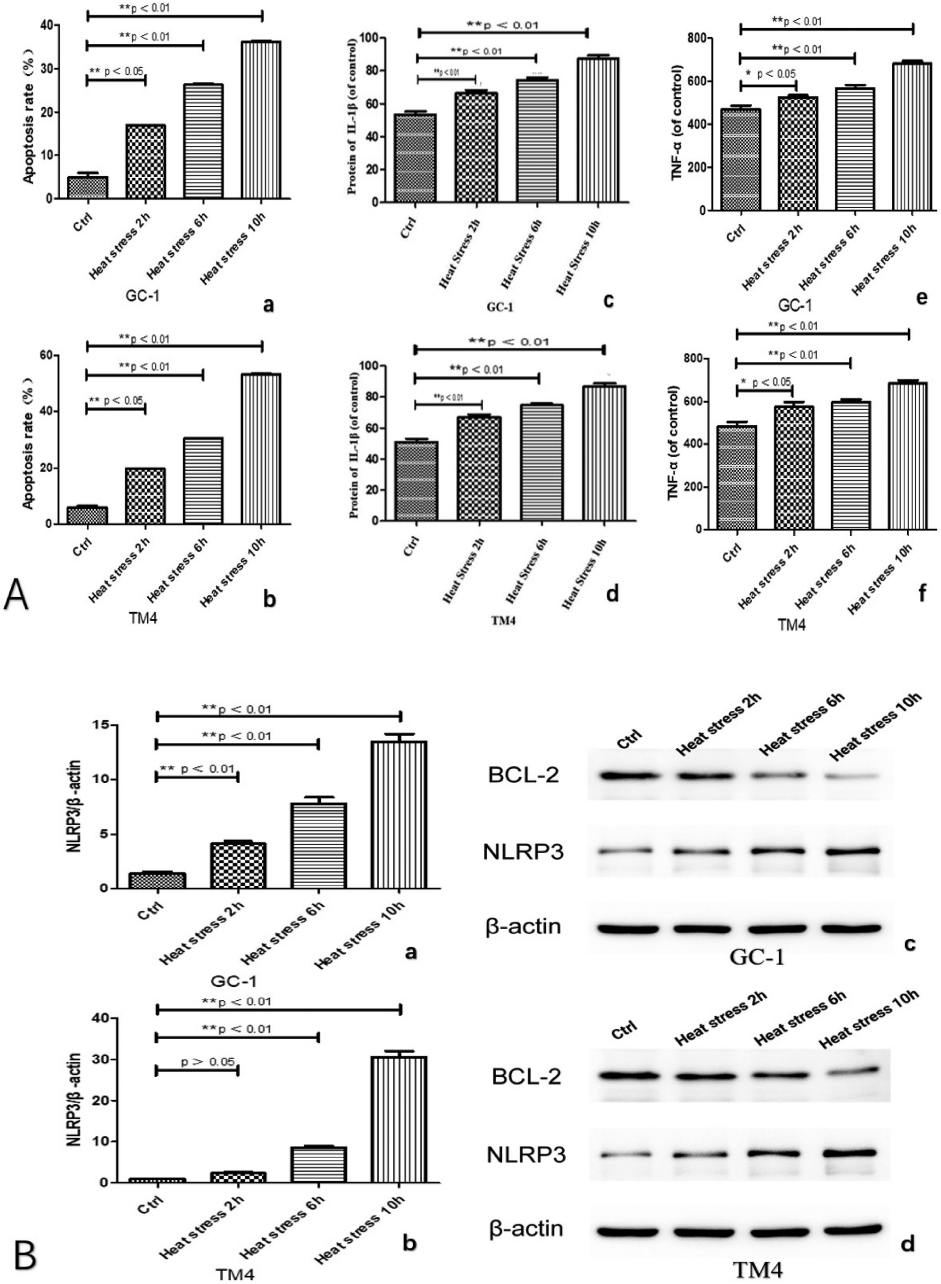
Both types of cells exhibited an increased apoptosis rate with prolonged exposure to heat stress **(A: a and b)**, accompanied by a corresponding rise in the concentration of inflammatory factors (TNF-α and IL-1β) **(A: c, d, e and f)**. Continuous heat stress led to heightened NLRP3 activity as indicated by PCR **(B: a and b)**, which was further confirmed by Western blotting (original blots), demonstrating elevated expression of NLRP3 in both cell types, with a more pronounced increase over time. Concurrently, the anti-apoptotic protein Bcl-2 displayed reduced expression in correlation with the escalating cell apoptosis **(B: c and d)**.

We observed an increase in the apoptosis rate of GC-1 and TM4 cells with prolonged exposure to heat stress (at 2, 6, 10 h, see Figures 3A: a and b, 5A). Correspondingly, the concentration of inflammatory factors (TNF-α and IL-1β) also increased (Figure 3A c–f). Continuous heat stress led to increased NLRP3 activity as indicated by PCR (Figure 3B: a and b), which was further confirmed by Western blotting, revealing elevated expression of NLRP3 in both cell types, with more pronounced expression over time. Additionally, the anti-apoptotic protein Bcl-2 exhibited reduced expression as cell apoptosis increased (Figure 3B: c and d).

### 3.3 Inhibition of NLRP3 expression reduces heat stress damage to both types of cells

**Figure 4:**
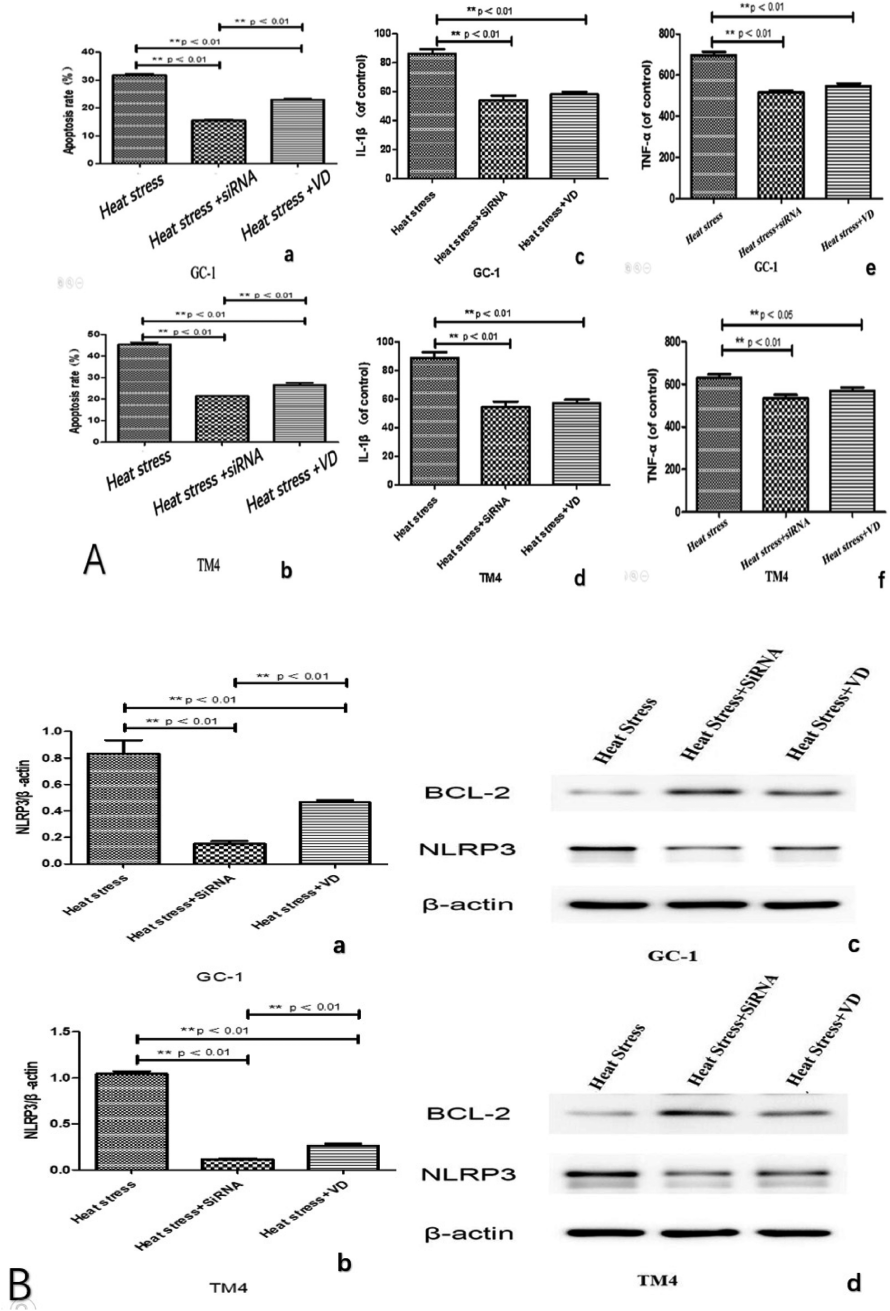
After 10 h of heat stress, compared to the heat stress group, the heat stress + VD group and heat stress + siRNA group exhibited reduced apoptosis **(A: a and b)** and lower levels of inflammatory factors **(A: c, d, e and f)**. NLRP3 activity as indicated by PCR in heat stress + VD group and heat stress + siRNA group is significantly lower **(B: a and b)**. The expression of the anti-apoptotic protein Bcl-2 in both groups was higher than that in the heat stress group, and the expression of NLRP3 protein was reduced **(B: c and d, original blots)**.

**Figure 5:**
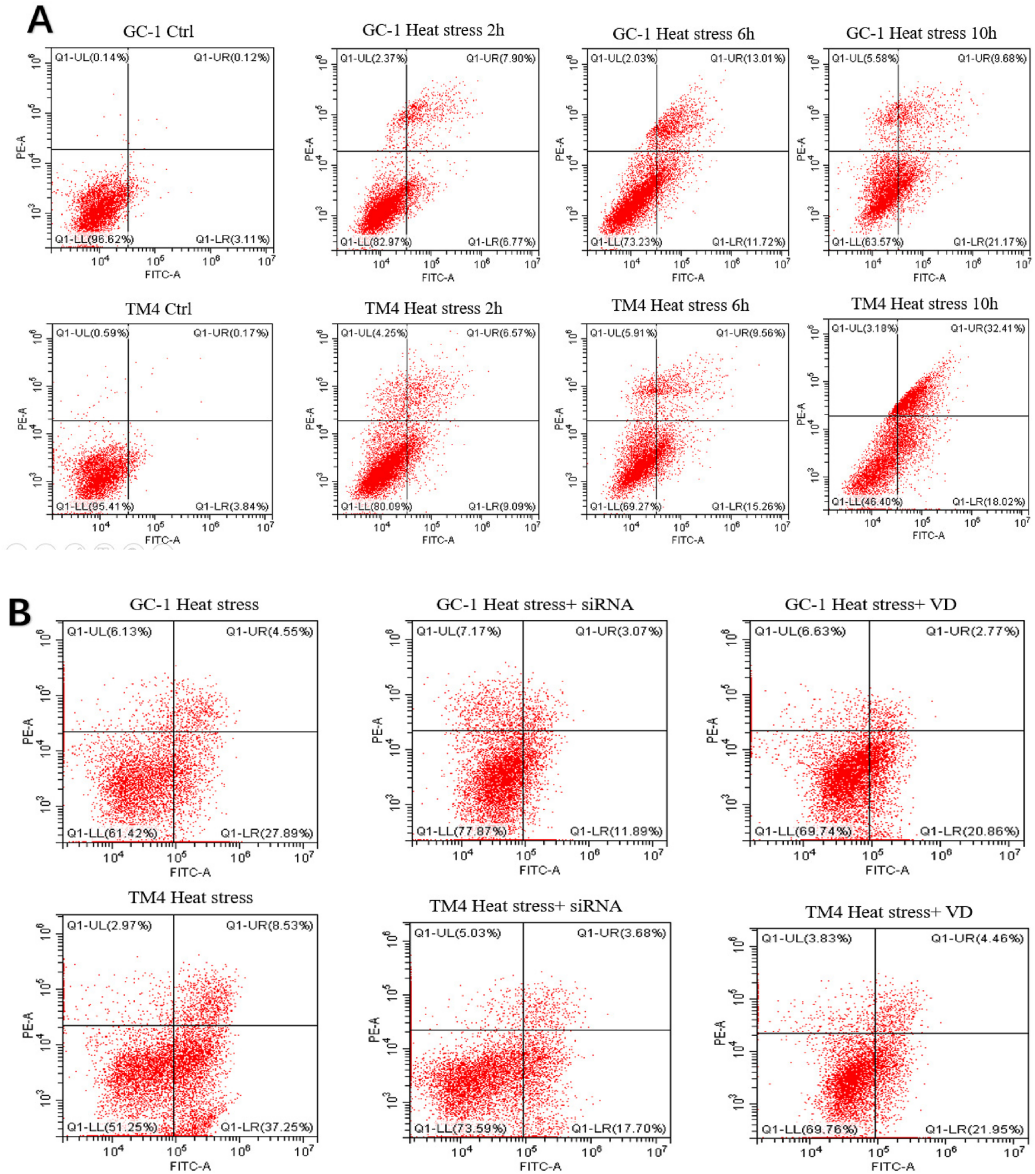
Dot plot images of flow cytometry: the results of apoptosis in two types of cells treated in different ways across different groups. **(A)** control group and heat stress at different time points. **(B)** control group, heat stress, heat stress + siRNA, and heat stress + VD group.

We observed significantly reduced cell apoptosis in both the VD + heat stress and siRNA + heat stress groups compared to the heat stress-only group (Figures 4A a and b, 5B). Additionally, we found lower levels of inflammatory factors (Figure 4A c–f) and a significant decrease in NLRP3 gene expression (Figure 4B a and b) in the VD + heat stress and siRNA + heat stress groups, with the most notable NLRP3 inhibition in the siRNA + heat stress group, showing statistically significant differences. Furthermore, BCL-2 protein expression increased while NLRP3 protein expression decreased (Figure 4 c and d).

## 4 Discussion

NLRP3 (NOD-like receptor family pyrin domain containing 3) is an inflammatory body-associated protein belonging to the NOD-like receptor (NLR) family. NLRP3 primarily plays a role in the immune system and specifically has an important function in the inflammatory process. Normally, the expression of the NLRP3 inflammasome is relatively low in cells. Its overexpression is involved in mediating the occurrence of various diseases, such as dermatitis, enteritis, diabetes, and pancreatitis, etc. (19, 20). In our experiment, NLRP3 showed weak expression in cryptorchid tissues, which is considered to be related to the fact that the cryptorchid tissues selected were testicular tissues of testicular regression syndrome, rather than true cryptorchid tissues. However, this is also the first time that the expression of NLRP3 has been proposed in cryptorchid tissues.

Varicocele induces testicular tissue hypoxia, elevated temperature, NLRP3 overexpression, and production of inflammatory mediators (IL-1β, TNF-α), impairing testicular function. Hajipour et al. experimented with resveratrol (RES) on varicocele rats; results showed RES inhibits NLRP3, reduces inflammation and apoptosis, alleviating varicocele complications (21). Hong et al. found in animal experiments that the phthalate derivative di-(2-ethylhexyl) phthalate (DEHP) causes oxidative stress response, activates the ROS/mTOR pathway, and triggers NLRP3 inflammasome activation in the testis, damaging germ cells and impairing testicular function (22). Sano et al. (23) have indicated that testicular cells have the potential to secrete IL-1β and TNF-α in an NLRP3 inflammasome-dependent manner, and that these testicular cell-derived cytokines may further exacerbate testicular function, leading to decreased fertility during infectious diseases. Cryptorchidism can also cause oxidative stress response due to high local temperature in the testis (24), and we speculate that cryptorchidism leads to oxidative stress that results in high expression of NLRP3, which produces a large amount of inflammatory factors, causing cellular damage that affects testicular function. Our experimental results also confirmed that with the prolongation of heat stress, the expression of NLRP3 in both types of cells increased, as did the levels of inflammatory factors, leading to cellular damage.

Sertoli cells (SCs) are the sole cells in the spermatogenic epithelium that directly interact with and provide support to the spermatogenic cells. They play a crucial role in forming the blood-testis-barrier (BTB) through their tight junctions, establishing an environment essential for spermatogenesis to occur smoothly and efficiently (25). It has indicated that SCs are vulnerable to various factors that can impact their function. For example, influences like temperature fluctuations and bisphenol A have been shown to diminish both the proliferative capacity and the expression of tight junction proteins in SCs, leading to disruptions in the blood-testis barrier and subsequently interfering with normal spermatogenesis (12, 26, 27). Studies have demonstrated that subjecting the testes to localized warm baths can trigger the dedifferentiation of testicular SCs in adult monkeys, resulting in the loss of supportive capabilities and ultimately halting spermatogenesis (28).

Our experiments likewise found an increase apoptosis in both types of cells with prolonged thermal stress. At the same time, we found that heat stress led to increased expression of inflammatory factors and increased NLRP3 activity. It suggests that heat stress leading to inflammatory response plays a key role in testicular cell damage.

Wang et al. conducted experimental animal studies and discovered that exposure to high temperatures initiated oxidative stress in testicular tissues, leading to negative impacts on the cells of porcine testicular tissues and the production of testosterone, consequently hindering testicular spermatogenesis (29).

The Bcl-2 gene is a key anti-apoptotic factor within its family (30). Inflammatory agents IL-1, TNF-α, and IFN-γ regulate the expression of Bcl-2 family proteins. An imbalance in inflammatory factor release disrupts the equilibrium between pro-apoptotic and anti-apoptotic proteins, leading to decreased Bcl-2 expression and resulting in cell apoptosis (31).

Numerous studies have been reported using vitamin D to inhibit NLRP3 activity for the treatment of related diseases. WU et al. found that vitamin D can activate the AMPK pathway and inhibit the mTOR pathway, thereby inhibiting NLRP3 inflammasome activation and attenuating cellular pyroptosis in β-cell dysfunction, which reduces the incidence of diabetes (32). Abdelrahman et al. revealed that calcitriol can improve non-alcoholic steatohepatitis by inhibiting the activation of NLRP3 inflammasomes in an autophagy-dependent manner (33). But whether VD can prevent and improve reproductive function in children with cryptorchidism is unknown. Our experimental results showed that both the VD + heat stress and siRNA + heat stress groups reduced NLRP3 expression and inflammatory factor secretion, leading to increased Bcl-2 expression and decreased apoptosis. We believe that NLRP3 mediates cell damage in cryptorchid testes, and that vitamin D can partially inhibit NLRP3 activation, reducing cellular inflammation and protecting against damage. Therefore, we speculate that NLRP3 may serve as a therapeutic target for protecting testicular tissue from heat stress.

## 5 Conclusions

Our experiment first discovered the expression of NLRP3 in cryptorchidism. The expression of NLRP3 in cryptorchidism leads to an increase in the concentration of inflammatory factors, playing an important role in the apoptosis of GC-1 and TM4 cells. Vitamin D can inhibit the expression of NLRP3 and reduce the levels of inflammatory factors, thus protecting GC-1 and TM4 cells from heat stress damage. This finding helps deepen the understanding of the molecular mechanisms behind testicular damage in cryptorchidism and provides innovative insights for clinical treatment of the condition. It also offers a theoretical basis for supplementing vitamin D in children with cryptorchidism awaiting treatment to protect testicular function.

There are still some limitations to our research: our findings are based on *in vitro* studies using cell lines, lacking animal experiments. The cryptorchid tissue used was not accurately defined, and the sample size was too small. Moving forward, we will expand our pathological specimens and conduct animal experiments to validate our *in vitro* results.

## Data Availability

The datasets presented in this study can be found in online repositories. The names of the repository/repositories and accession number(s) can be found in the article/Supplementary Material.
