# Supplementary figures and images for: Vitamin D protects spermatogonia and Sertoli cells from heat stress damage by inhibiting NLRP3
Source: Front Pediatr. 2025 Jan 7;12:1495310. doi: 10.3389/fped.2024.1495310 (PMC11747411; doi:10.3389/fped.2024.1495310)

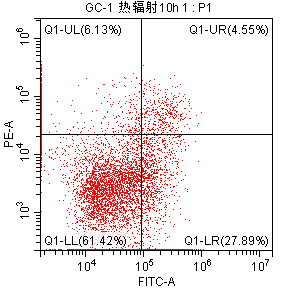


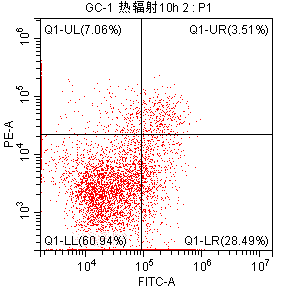


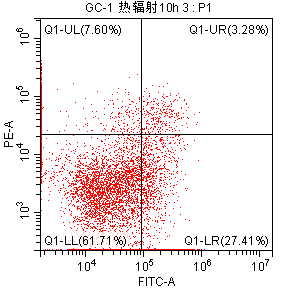


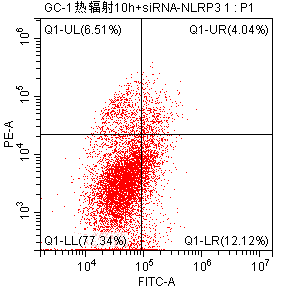


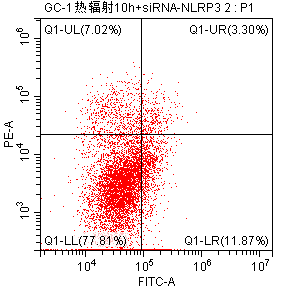


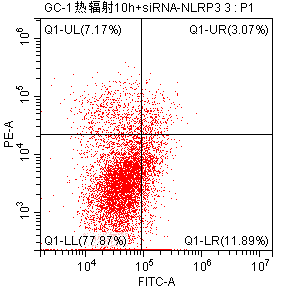


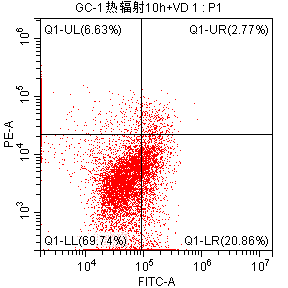


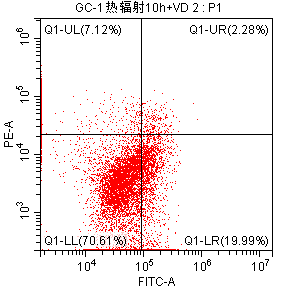


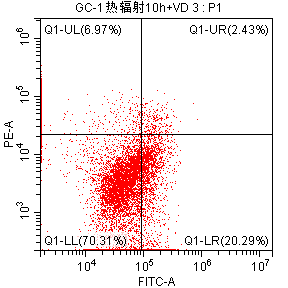

Supplement: Supplementary file 2 [file Table4.docx]

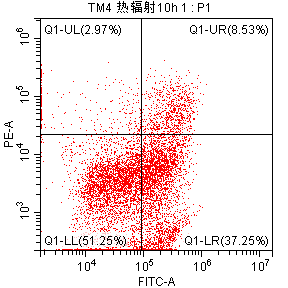


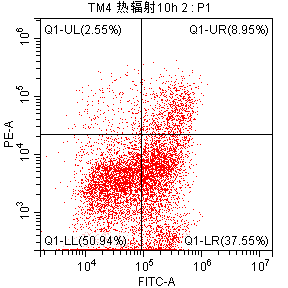


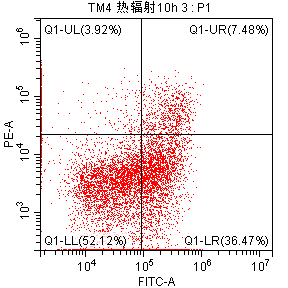


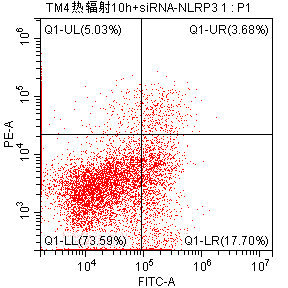


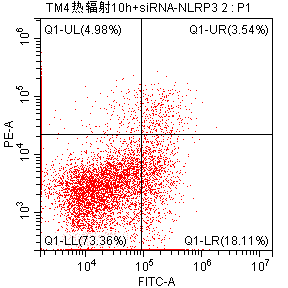


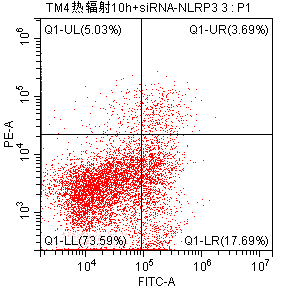


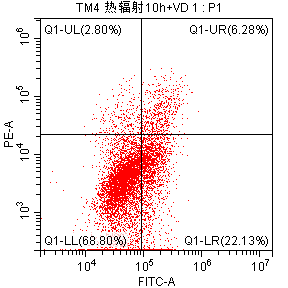


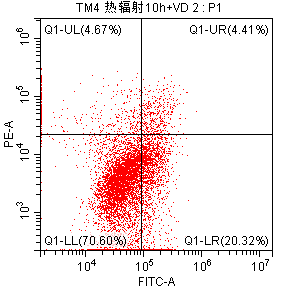


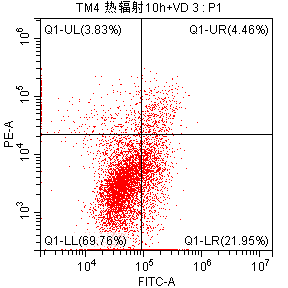

Supplement: Supplementary file 3 [file Table5.docx]

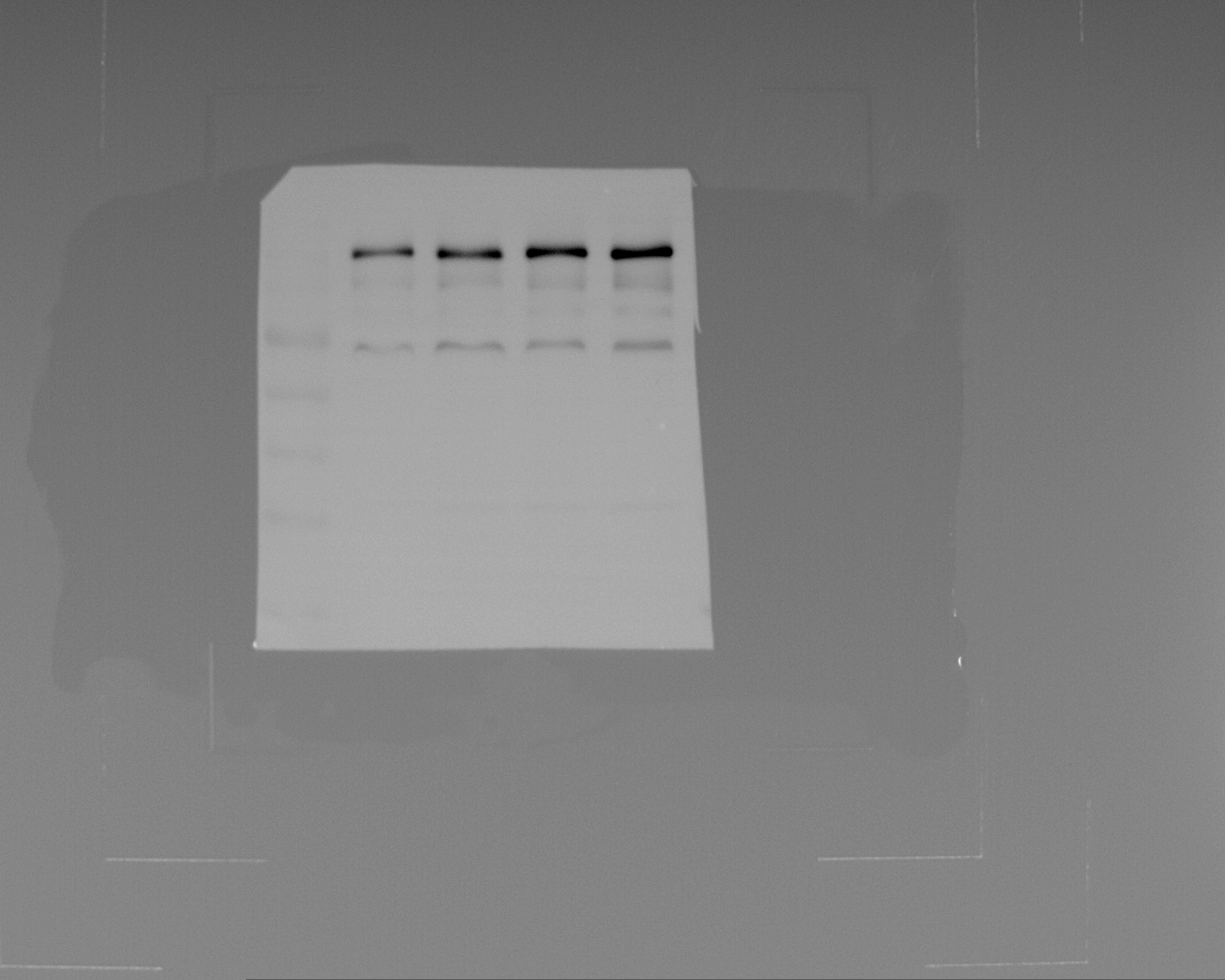

Supplement: Supplementary file 5 [file Image5.tif]

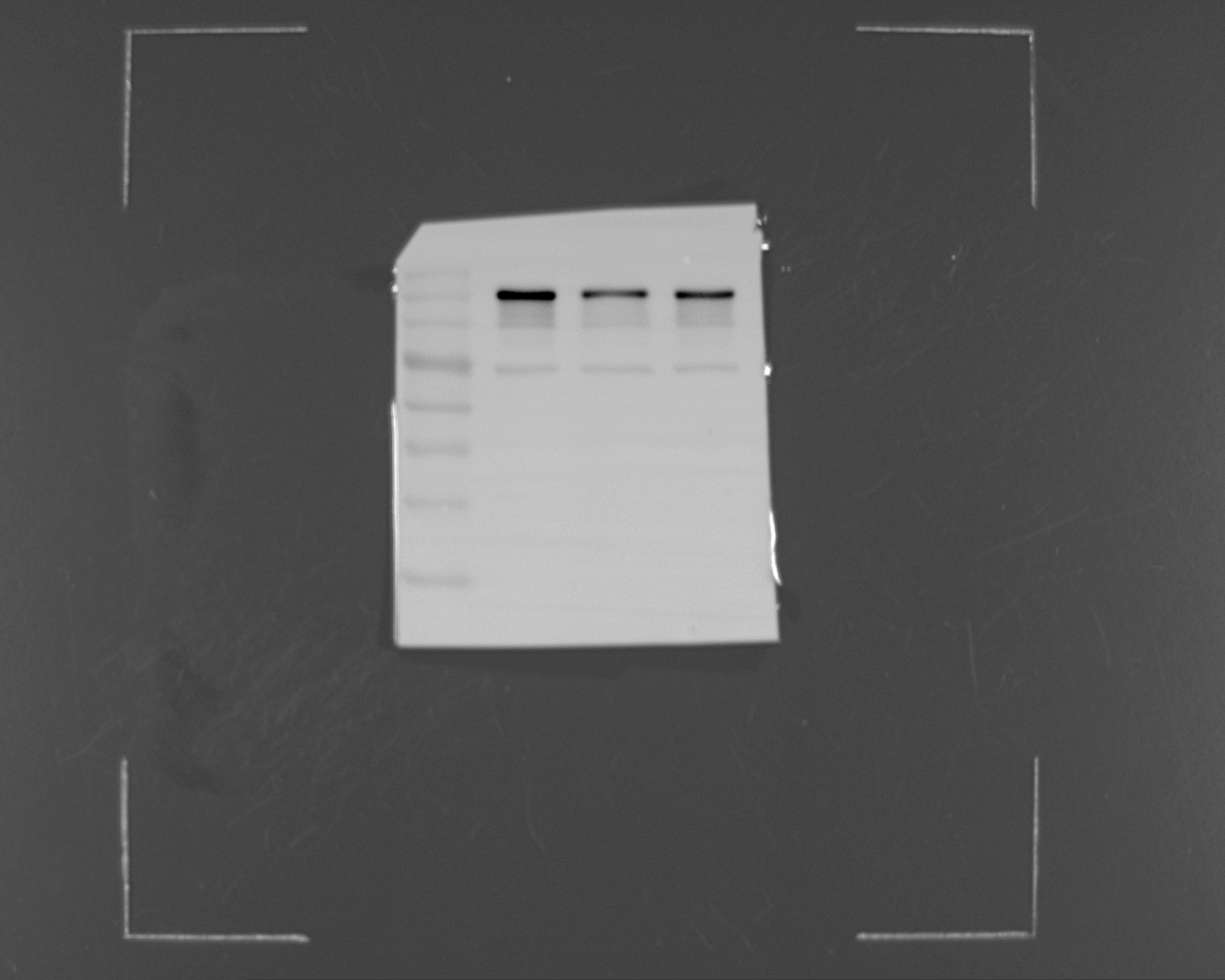

Supplement: Supplementary file 6 [file Image6.tif]

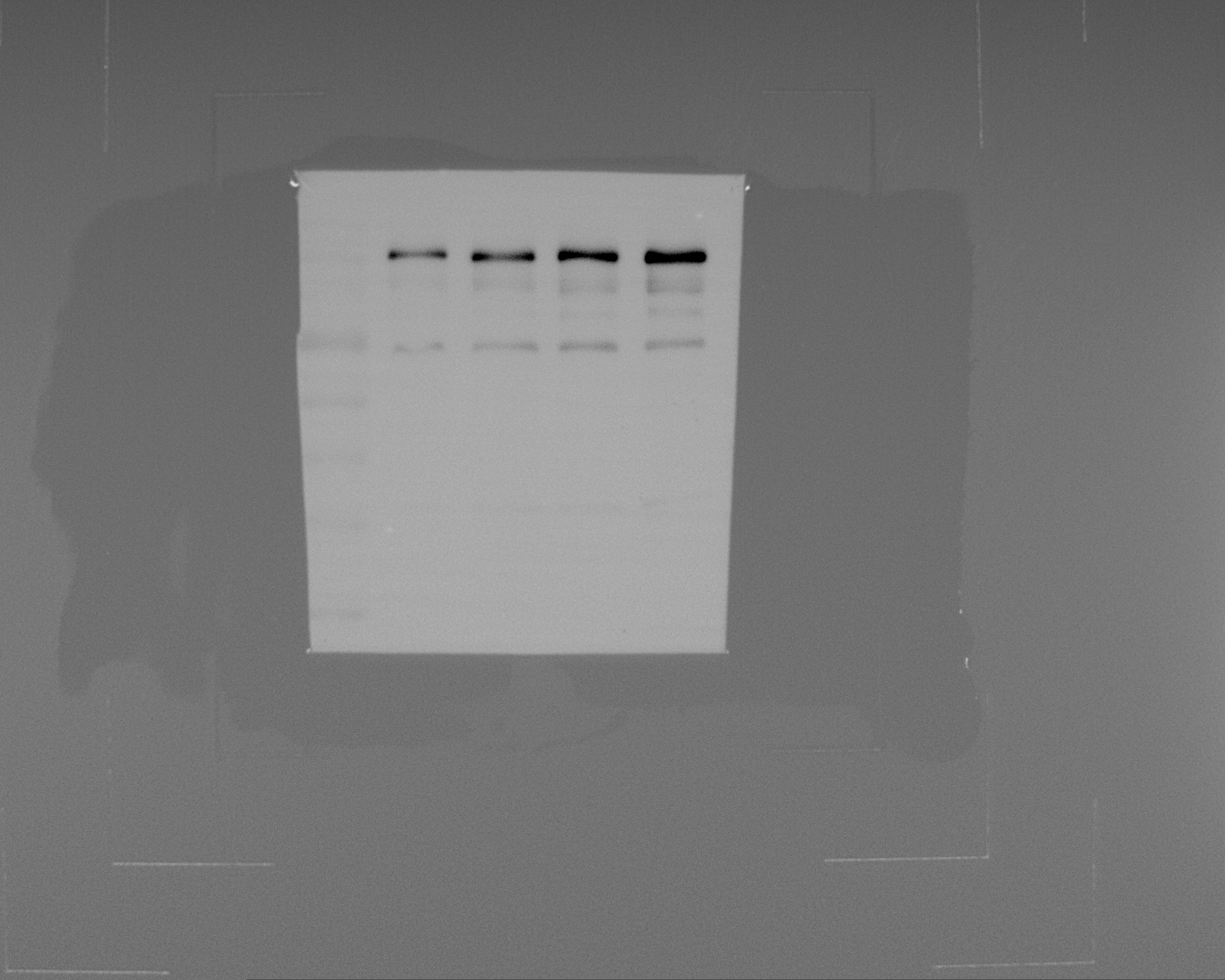

Supplement: Supplementary file 7 [file Image7.tif]

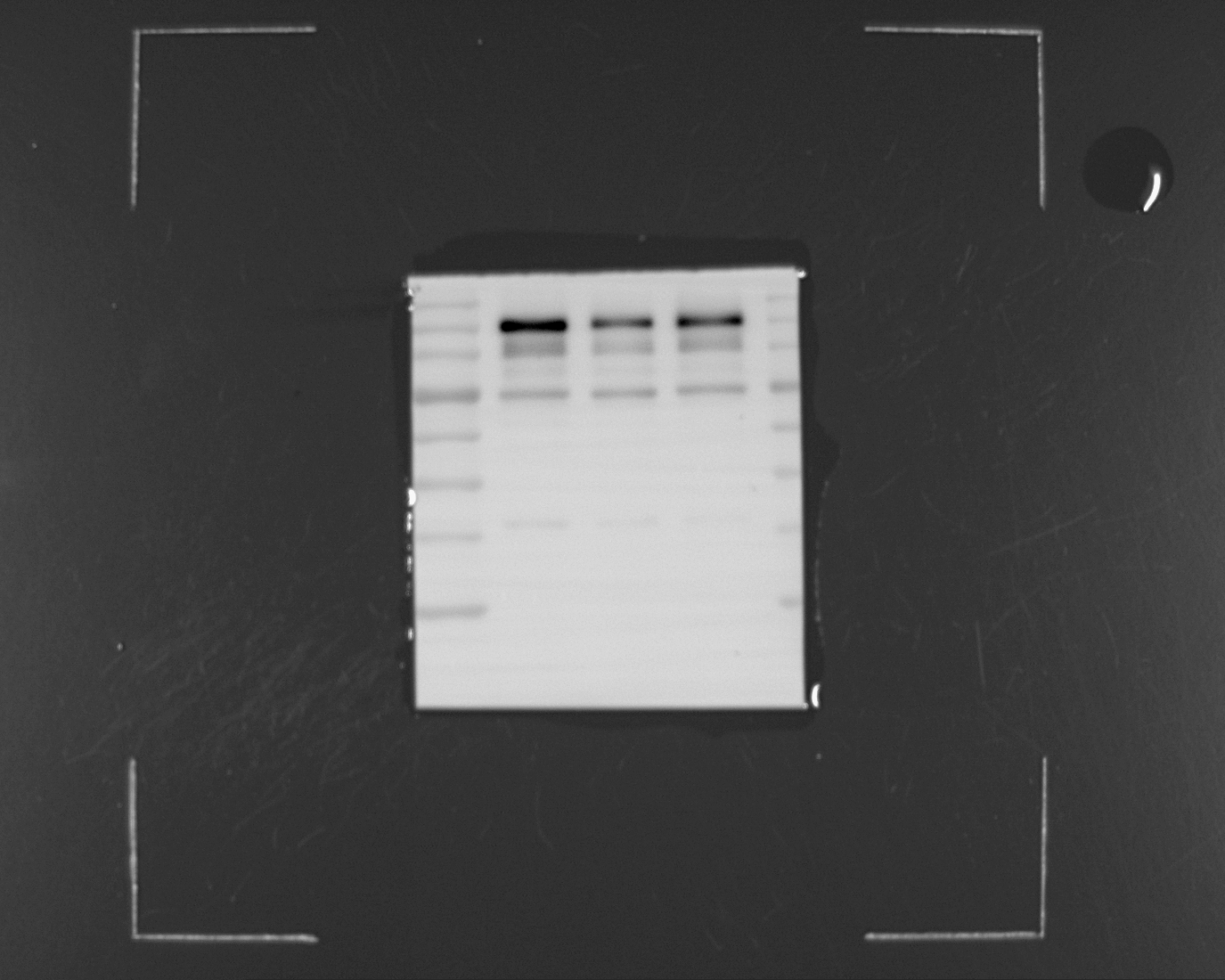

Supplement: Supplementary file 8 [file Image8.tif]

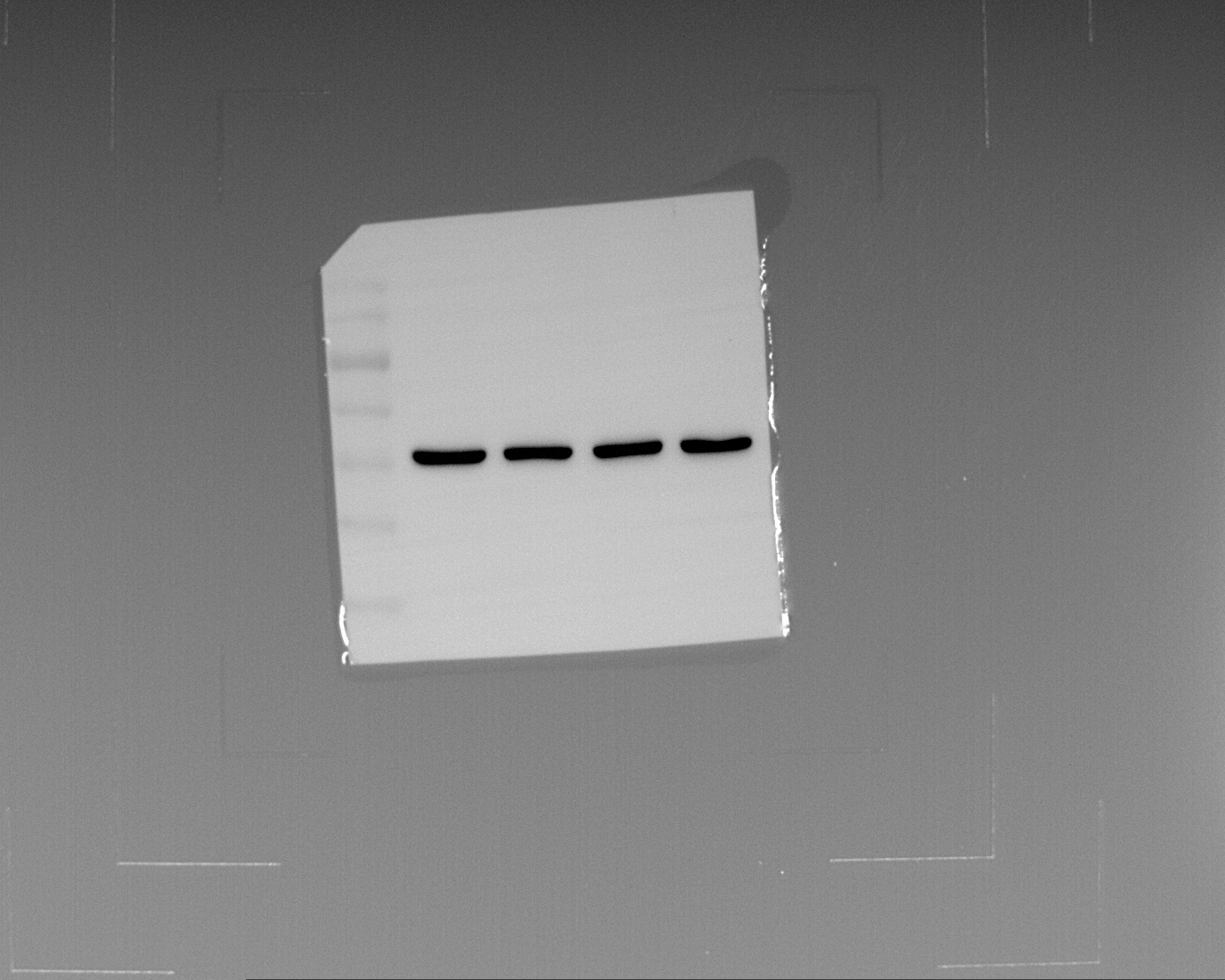

Supplement: Supplementary file 9 [file Image9.tif]

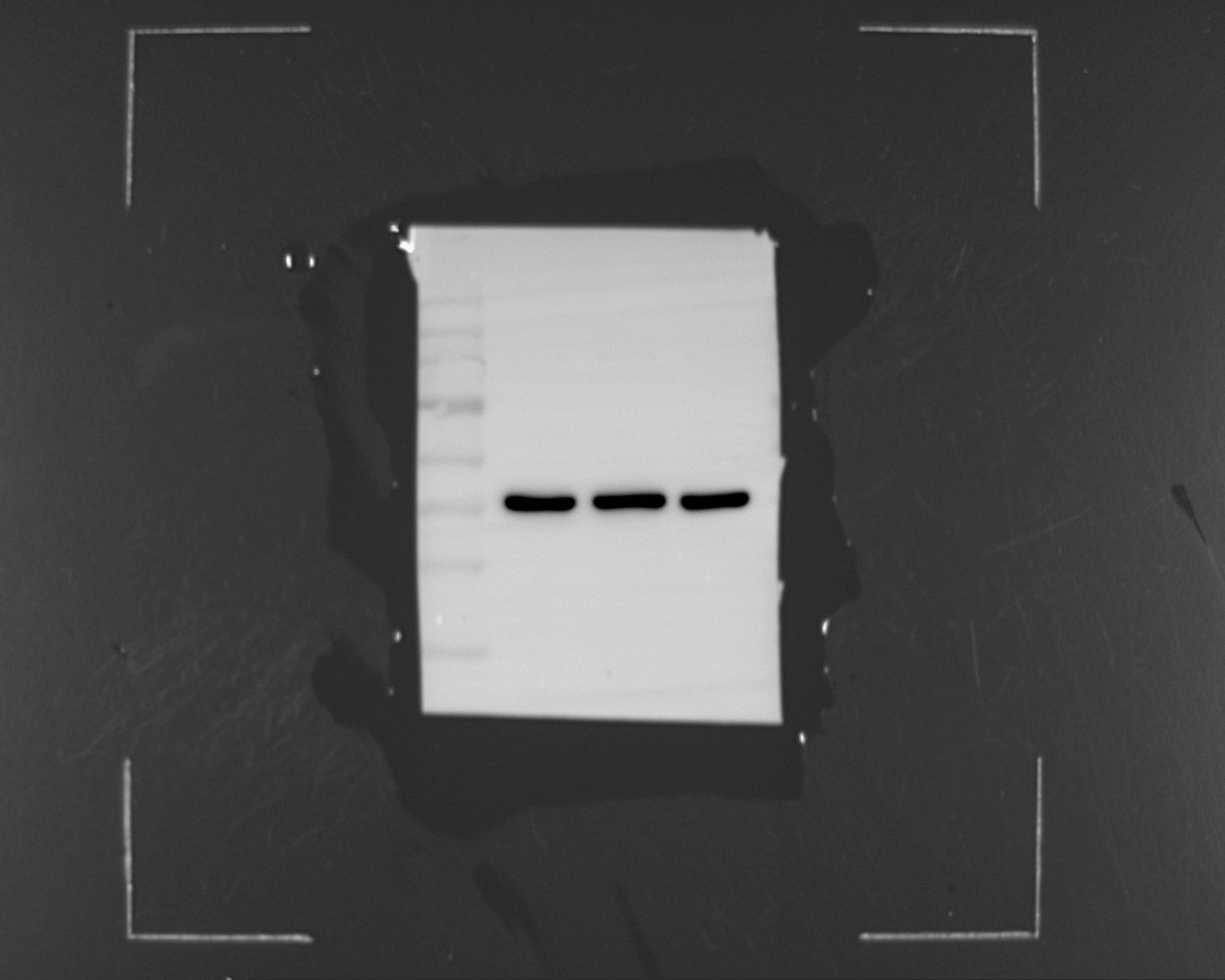

Supplement: Supplementary file 10 [file Image10.tif]

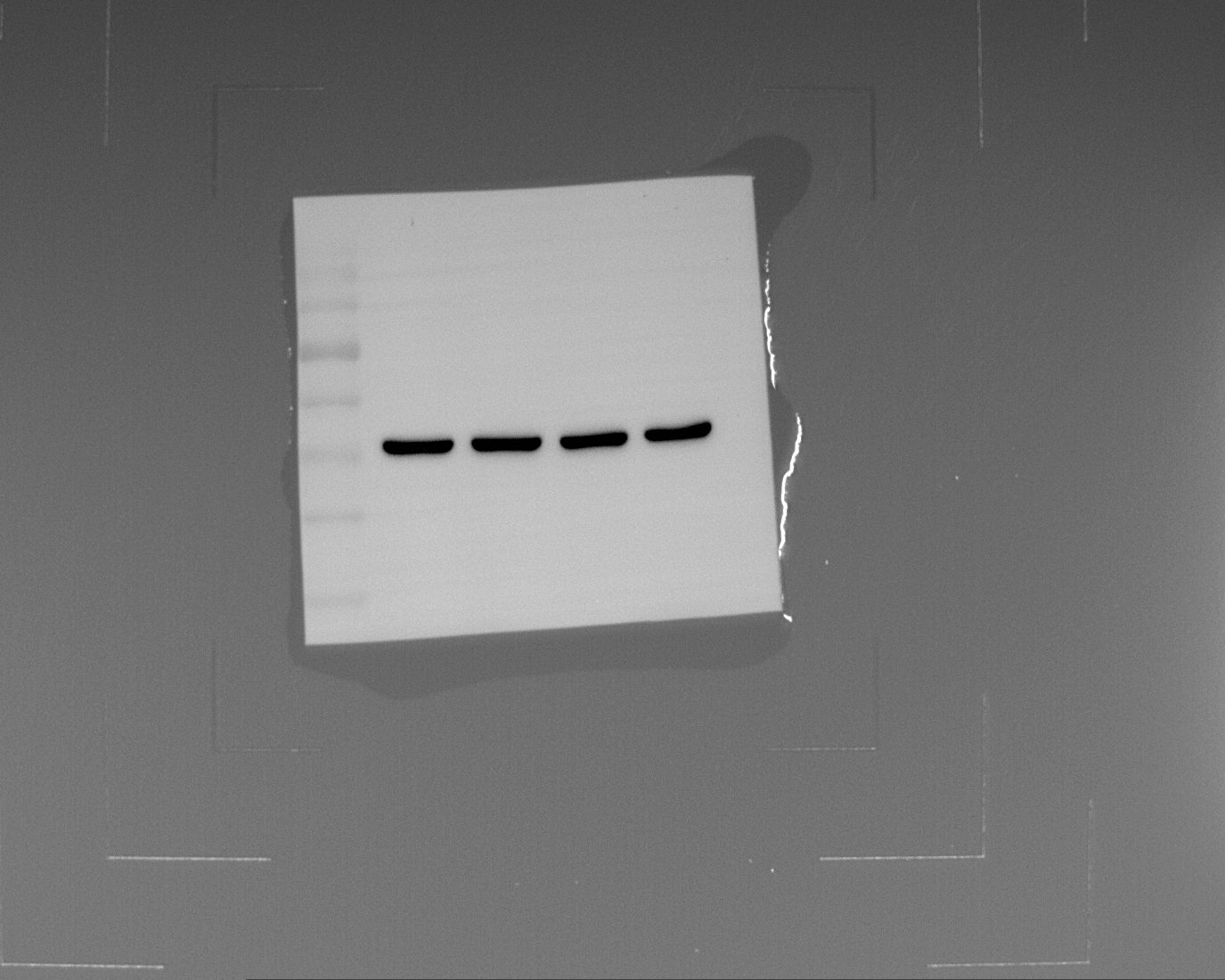

Supplement: Supplementary file 11 [file Image11.tif]

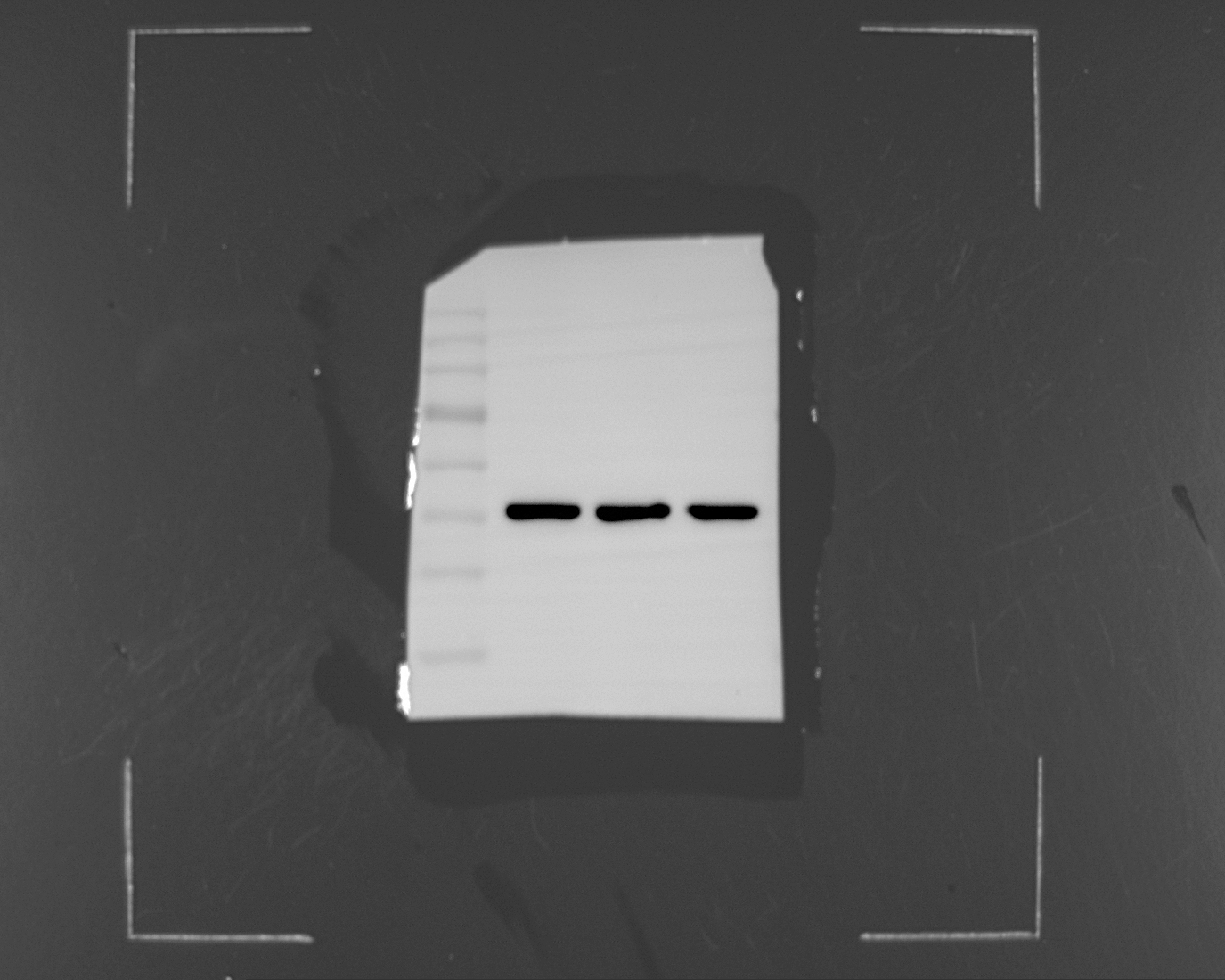

Supplement: Supplementary file 12 [file Image12.tif]

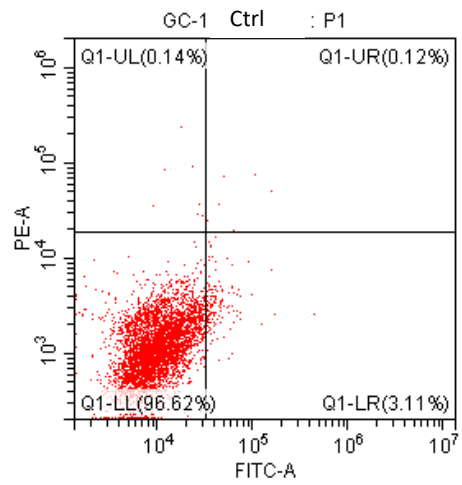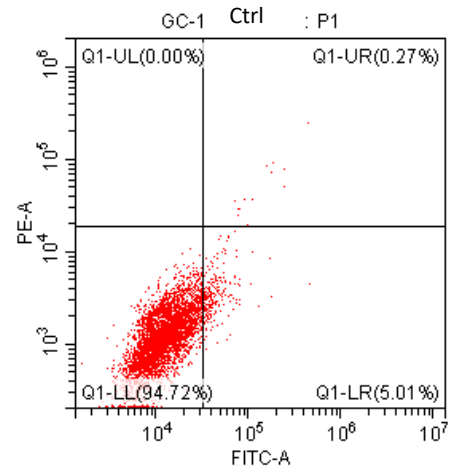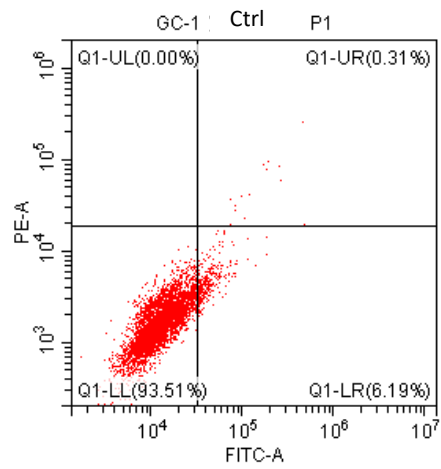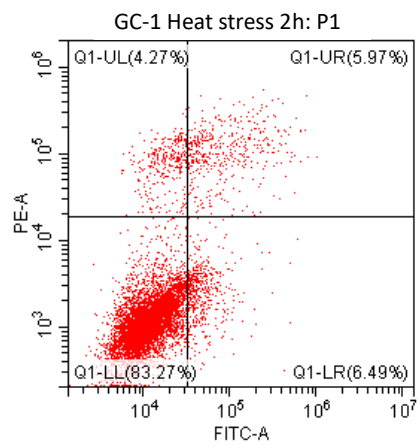

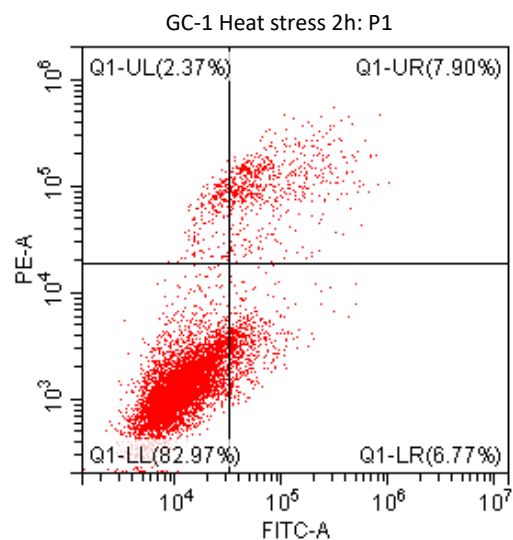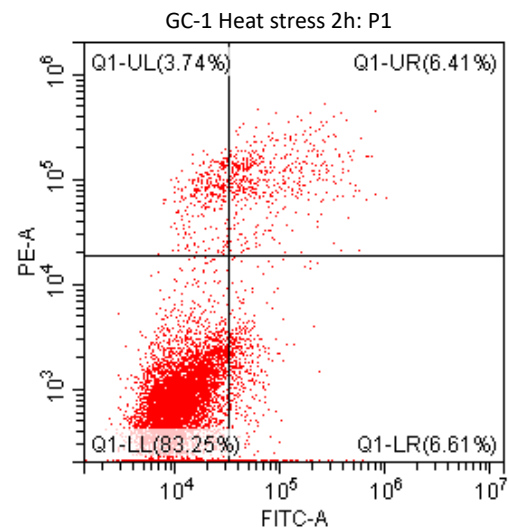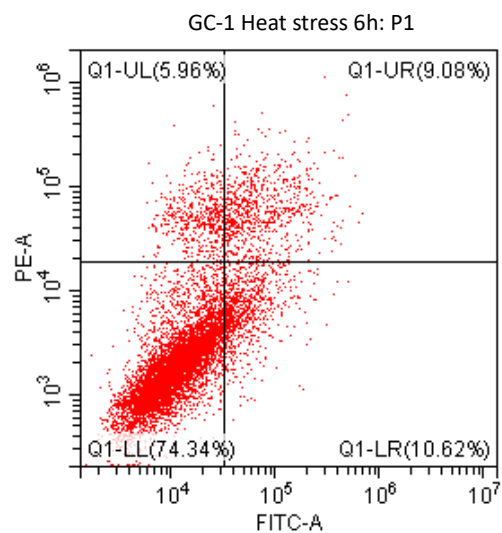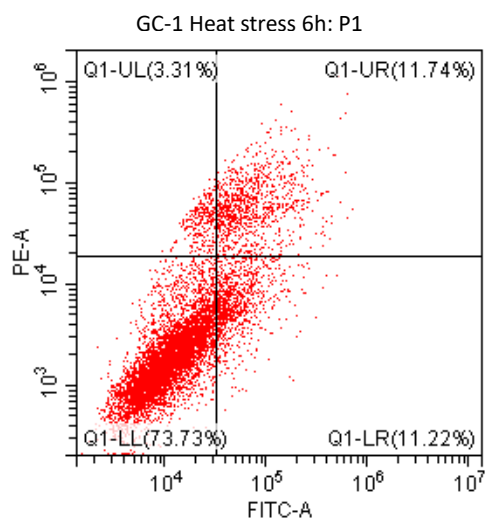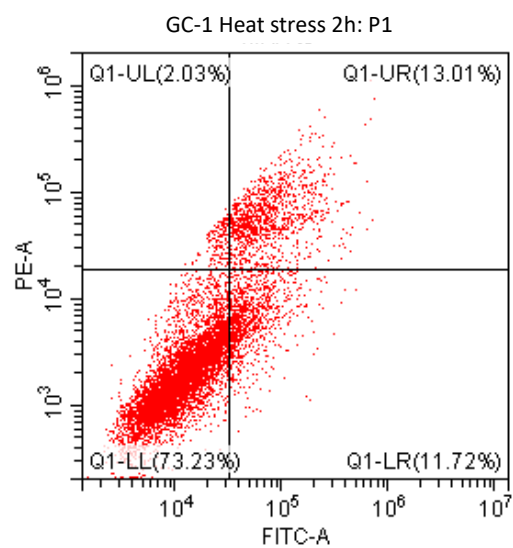

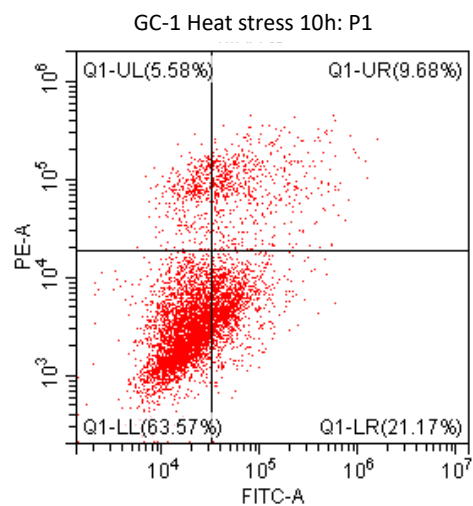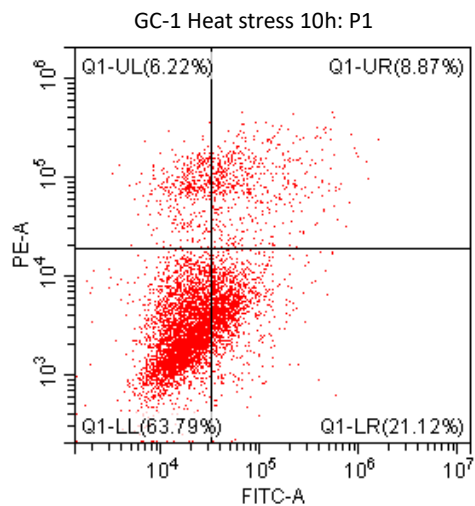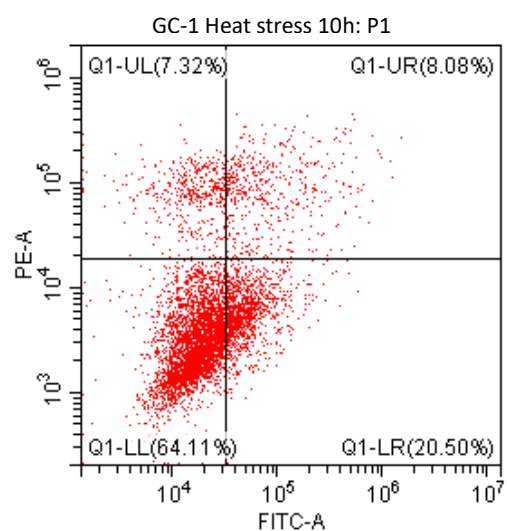

Supplement: Supplementary file 13 [file Image1.pdf]

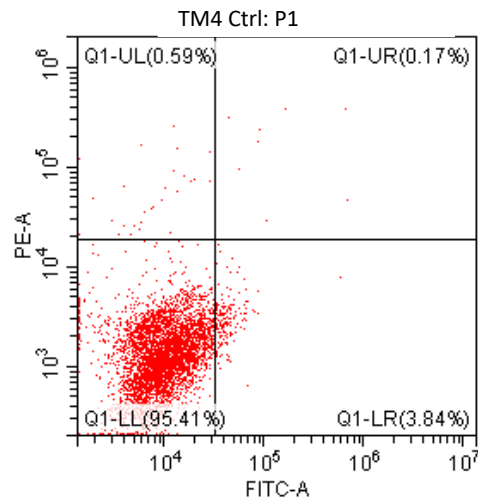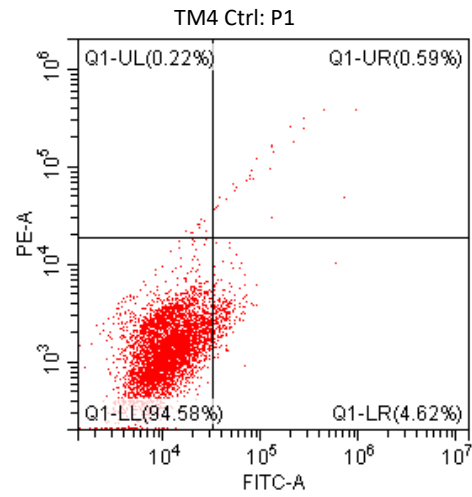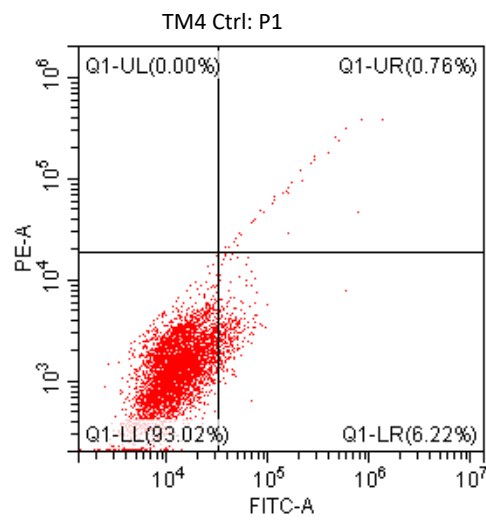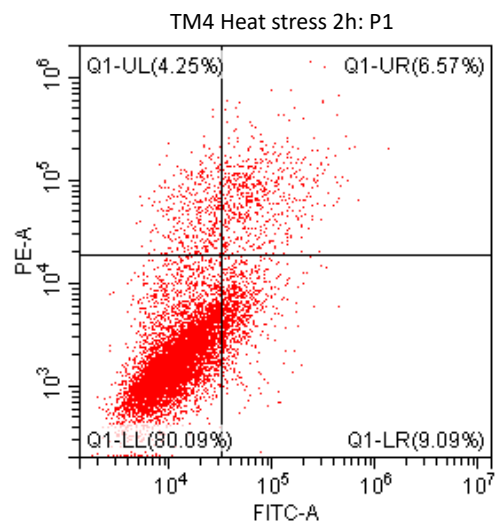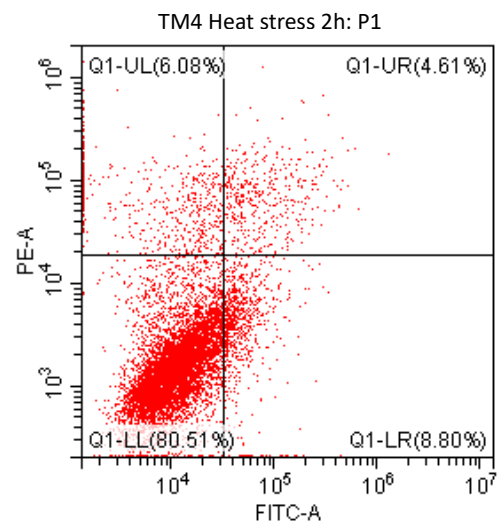

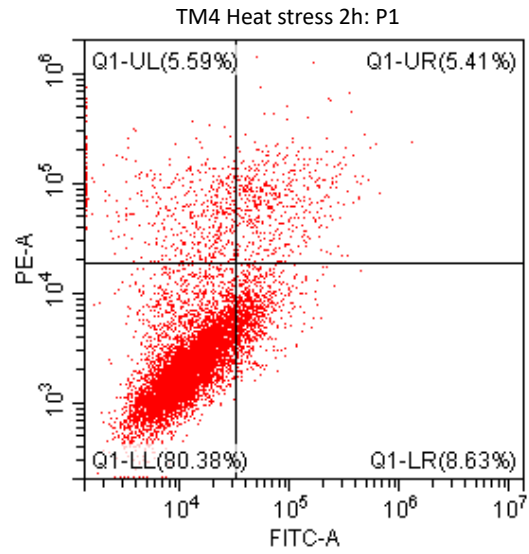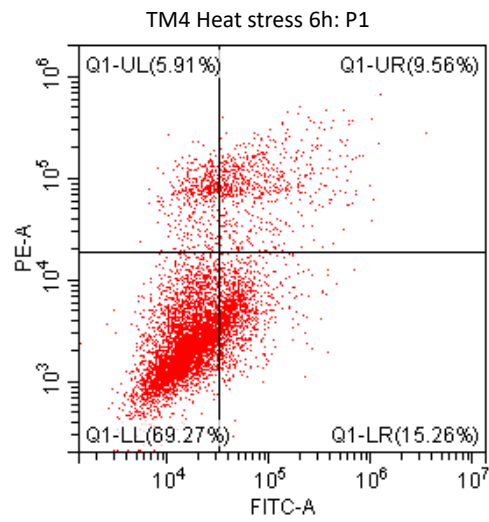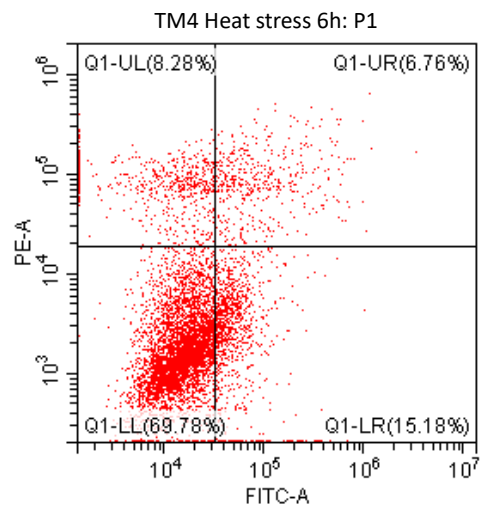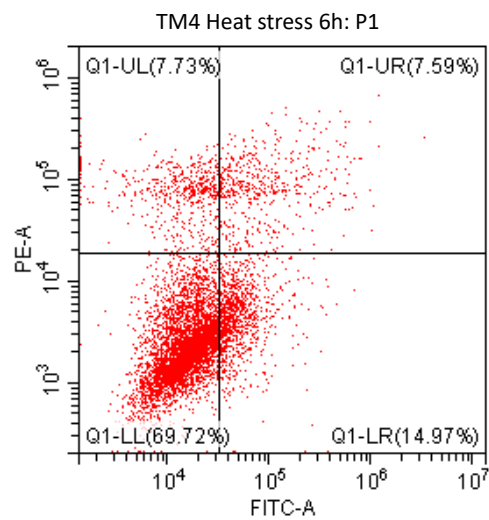

TM4 Heat stress 10h: P1

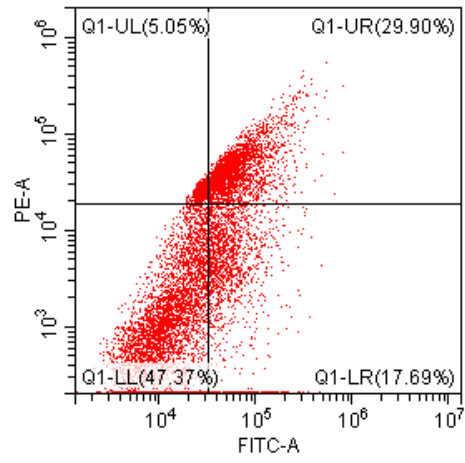

TM4 Heat stress 10h: P1

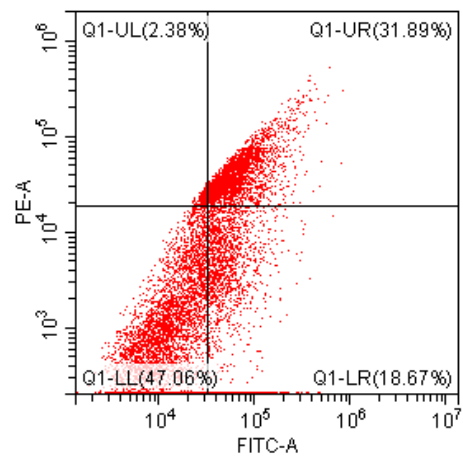

TM4 Heat stress 10h: P1

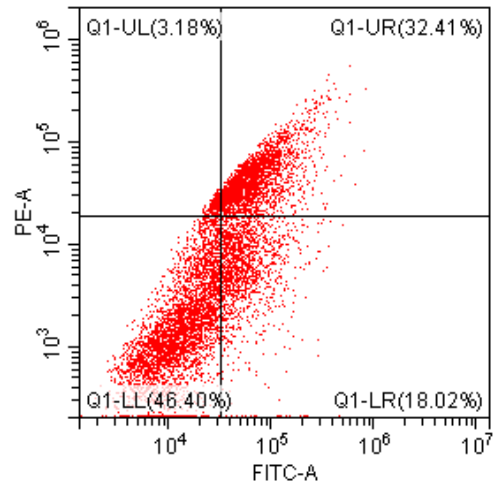

Supplement: Supplementary file 14 [file Image2.pdf]
