# Supplementary material for: Vitamin D protects spermatogonia and Sertoli cells from heat stress damage by inhibiting NLRP3
Source: Front Pediatr. 2025 Jan 7;12:1495310. doi: 10.3389/fped.2024.1495310 (PMC11747411; doi:10.3389/fped.2024.1495310)

GC-1 Heat stress 10h 1： P1

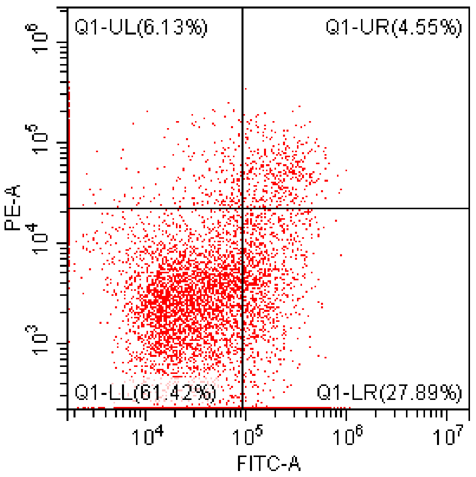

GC-1 Heat stress 10h 2： P1

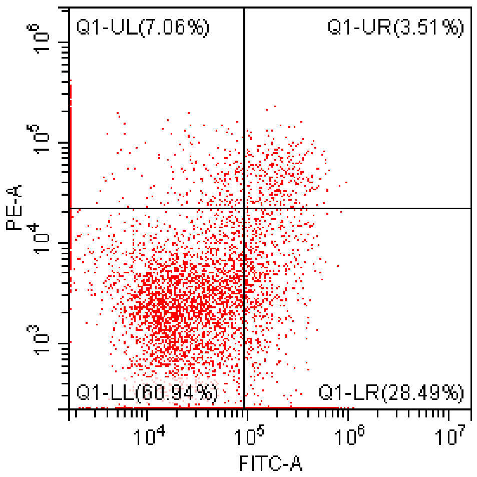

GC-1 Heat stress 10h 3： P1

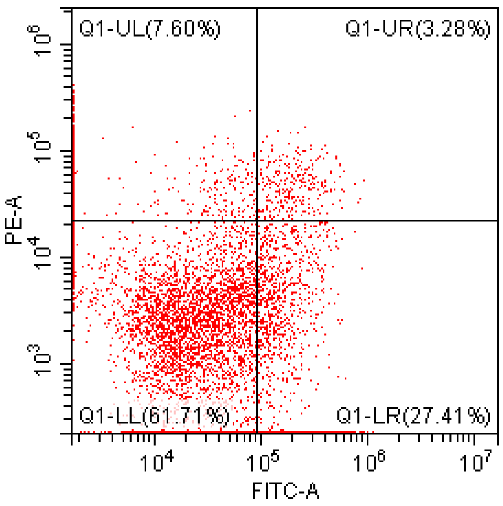

GC-1 Heat stress10h + siRNA-NLRP3 1： P1

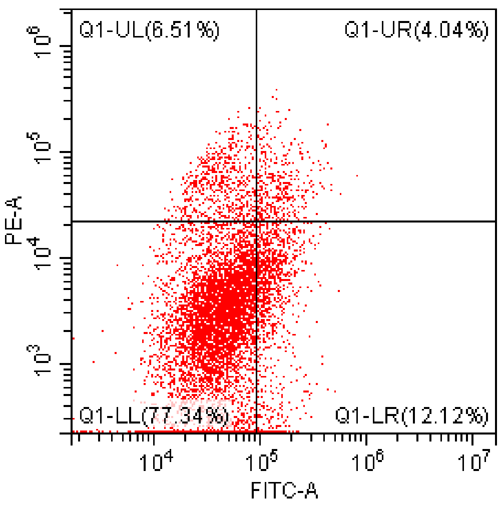

GC-1 Heat stress10h + siRNA-NLRP3 2： P1

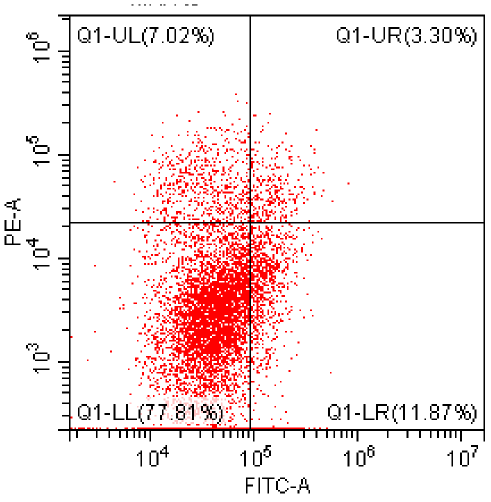

GC-1 Heat stress10h + siRNA-NLRP3 3：P1

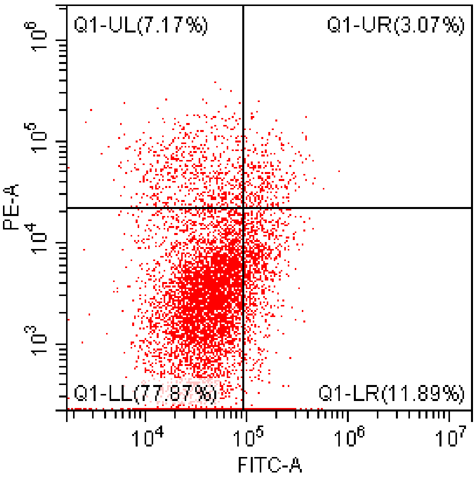

GC-1 Heat stress10h +VD 1：P1

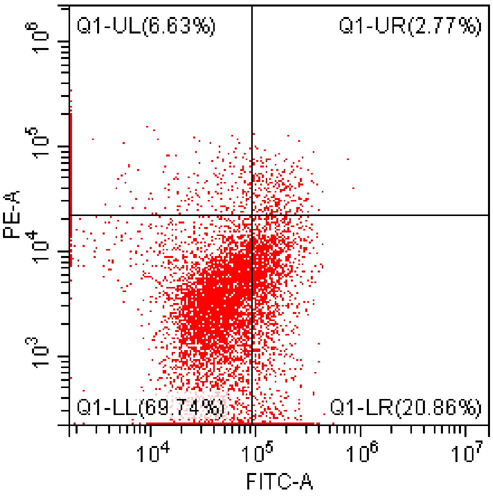

GC-1 Heat stress10h +VD 2：P1

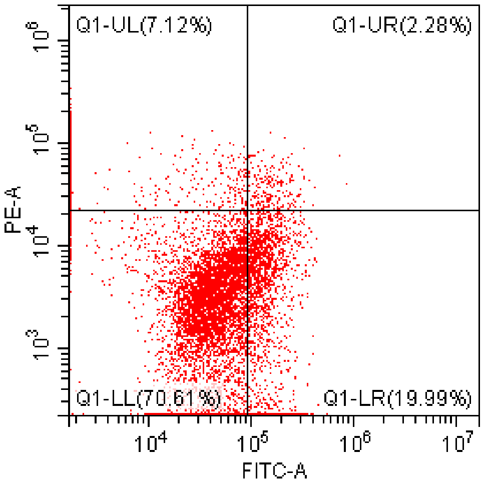

GC-1 Heat stress10h +VD 3：P1

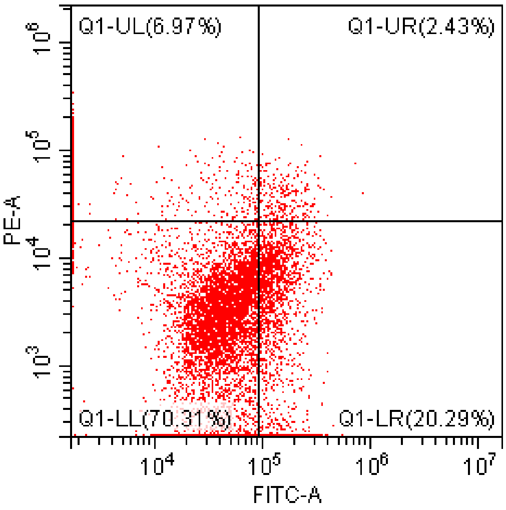

Supplement: Supplementary file 15 [file Image3.pdf]
