# Supplementary material for: Vitamin D protects spermatogonia and Sertoli cells from heat stress damage by inhibiting NLRP3
Source: Front Pediatr. 2025 Jan 7;12:1495310. doi: 10.3389/fped.2024.1495310 (PMC11747411; doi:10.3389/fped.2024.1495310)

TM4 Heat stress 10h 1: P1

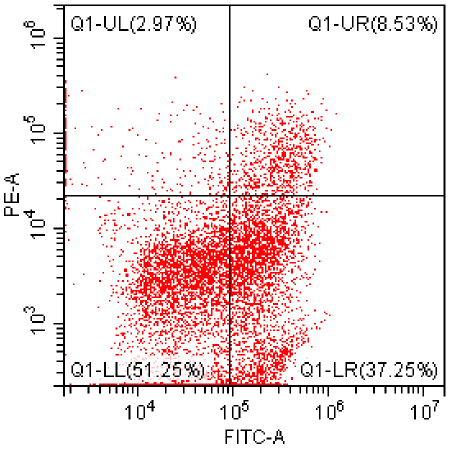


TM4 Heat stress 10h 2: P1

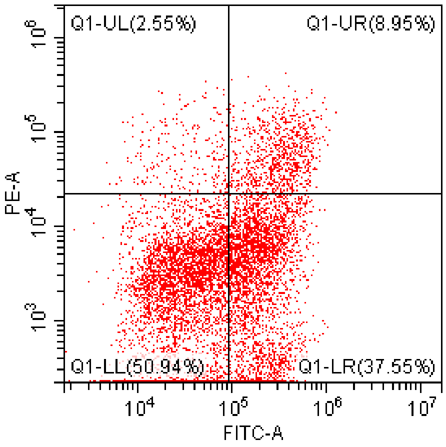


TM4 Heat stress 10h 3: P1

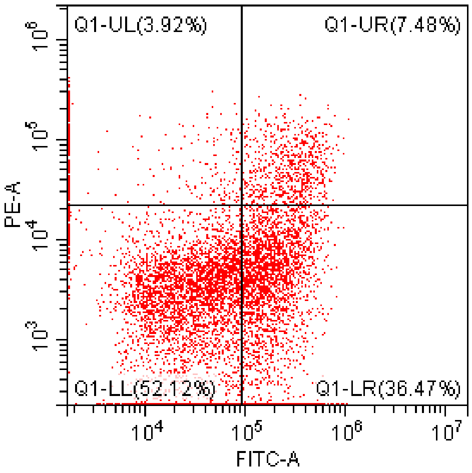


TM4 Heat stress 10h+siRNA-NLRP3 1: P1

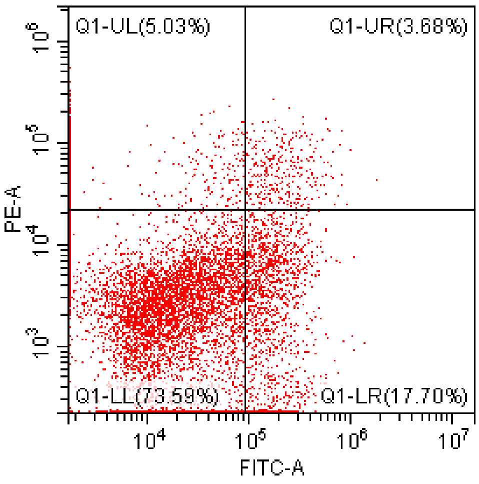


TM4 Heat stress 10h+siRNA-NLRP3 2: P1

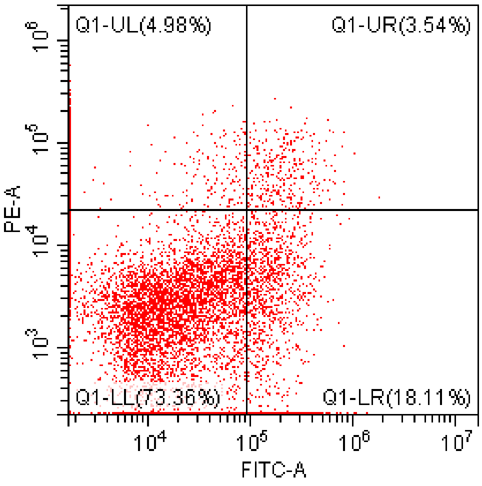

TM4 Heat stress 10h+siRNA-NLRP3 3: P1

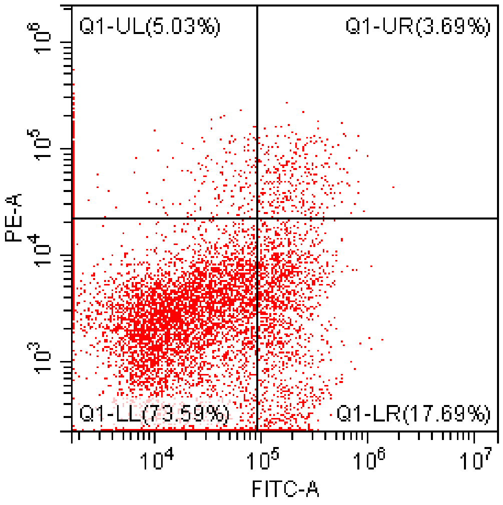


TM4 Heat stress 10h+VD 1: P1

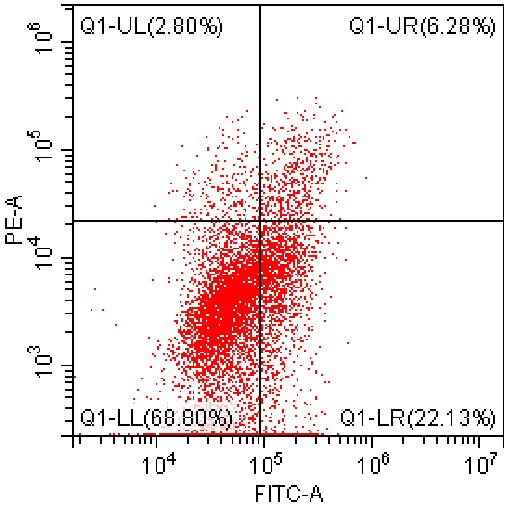


TM4 Heat stress 10h+VD 2: P1

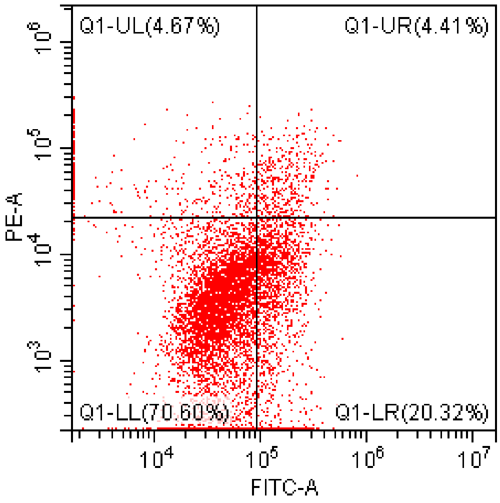


TM4 Heat stress 10h+VD 3: P1

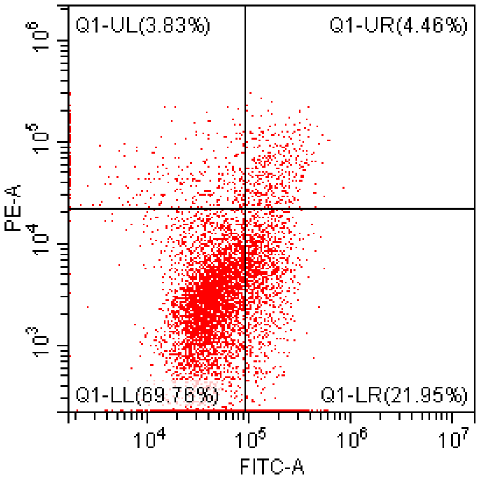

Supplement: Supplementary file 16 [file Image4.pdf]
